# Supramolecular Polymer Co-Assembled Multifunctional Chiral Hybrid Hydrogels with Adhesive, Self-Healing and Antibacterial Properties

**DOI:** 10.3390/gels10080489

**Published:** 2024-07-24

**Authors:** Zakia Riaz, Sravan Baddi, Fengli Gao, Xiaxin Qiu, Chuanliang Feng

**Affiliations:** State Key Lab of Metal Matrix Composites, Shanghai Key Laboratory for Molecular Engineering of Chiral Drugs, School of Materials Science and Engineering, Shanghai Jiaotong University, Dongchuan Rd 800, Shanghai 200240, China; zakia_riaz@sjtu.edu.cn (Z.R.); fengligao123@sjtu.edu.cn (F.G.); xiaxinqiu@sjtu.edu.cn (X.Q.)

**Keywords:** hybrid hydrogel, co-assembly, self-healing, adhesive, antibacterial

## Abstract

Amino acid-derived self-assembled nanofibers comprising supramolecular chiral hydrogels with unique physiochemical characteristics are highly demanded biomaterials for various biological applications. However, their narrow functionality often limits practical use, necessitating the development of biomaterials with multiple features within a single system. Herein, chiral co-assembled hybrid hydrogel systems termed LPH-EGCG and DPH-EGCG were constructed by co-assembling L/DPFEG gelators with epigallocatechin gallate (EGCG) followed by cross-linking with polyvinyl alcohol (PVA) and hyaluronic acid (HA). The developed hybrid hydrogels exhibit superior mechanical strength, self-healing capabilities, and adhesive properties, owing to synergistic non-covalent interactions. Integrating hydrophilic polymers enhances the system’s capacity to demonstrate favorable swelling characteristics. Furthermore, the introduction of EGCG facilitated the hybrid gels to display notable antibacterial properties against both Gram-positive and Gram-negative bacterial strains, alongside showcasing strong antioxidant capabilities. In vitro investigation demonstrated enhanced cell adhesion and migration with the LPH-EGCG system in comparison to DPH-EGCG, thus emphasizing the promising prospects of these hybrid hydrogels in advanced tissue engineering applications.

## 1. Introduction

Peptide- and amino acid derivative-based chiral self-assembled nanofibers constituting supramolecular hydrogels exhibit distinct physicochemical attributes and closely resemble the extracellular matrix (ECM) [1,2]. These types of biomaterials hold immense potential for diverse biological applications, including drug delivery, wound healing, and tissue engineering. However, their narrow functionality often limits their practical applications. Therefore, it is highly demanding to develop such types of biomaterials through a facile preparation approach that holds multiple features within a single system and further expands their potential scope in various biomedical domains [3,4]. In recent times, there has been a surge of scientific interest in multi-component hybrid hydrogels due to their vast potential for diverse applications in biological contexts [5,6]. These hybrid systems not only aim to address and resolve issues associated with existing single-component formulations but also seek to broaden the scope of application possibilities [7,8]. By synthetically integrating supramolecular hydrogels with a diverse array of components, such as molecules and polymers, biomimetic hybrid hydrogels can be fabricated to enhance the intrinsic merits of each constituent [9,10,11]. Such hybrid systems exhibit multifunctional properties encompassing adhesion, self-healing, biocompatibility, antibacterial, and antioxidant capabilities within a unified framework, effectively eliminating the limitations associated with existing supramolecular gelators [12,13].

Supramolecular hybrid hydrogels are at the forefront of materials science, with their multifunctional properties making them highly suitable for a wide range of biomedical applications including drug delivery, tissue engineering, wound healing, and diagnostics [12,14]. Ongoing research is focused on developing new synthesis methods, exploring novel applications, and improving the performance of these hybrid materials for their applications in the biomedical arena [15]. In recent years, significant advancements in hybrid gel systems have been documented in the literature. One notable example is the work by Wei Gong et al., who developed a hybrid cross-linked network hydrogel (GA-AG) utilizing agarose and gallic acid. This hydrogel demonstrated high viscosity, self-healing capabilities, thermal stability, and efficient antibacterial activity, proving effective in promoting wound healing [16]. Similarly, Qigong et al. reported a supramolecular-polymeric hybrid hydrogel system that combines a co-assembled DBS-COOH/Naproxen network (DBS/NAP) with a polymeric gelatin (G) network incorporating quaternary ammonium groups (Q) and phenylboronic acid group (PBA)-grafted chitosan (QCS-PBA). This system enhances wound healing by increasing the mechanical strength of the drug-based supramolecular hydrogel and introducing potent antibacterial properties through the Q groups [17]. Despite these advancements, certain limitations persist in the reported hydrogel systems, including inadequate adhesion to wound surfaces and insufficient mechanical properties. In addition, these systems lack chiral characteristics, which are crucial for regulating cellular activities such as differentiation, adhesion, and proliferation within hydrogels, essential for tissue regeneration [18]. To address the shortcomings of the existing hybrid hydrogels, the development of chiral hybrid hydrogels is crucial. The superiority of chiral gels over achiral gels lies in their enhanced selectivity, controlled supramolecular assembly, tailored biological interactions, unique optical properties, and potential for sustainable applications. These advantages make chiral gels a promising class of materials for a wide range of advanced technologies and biomedical applications. Chiral gels show promise in biological and biomedical applications due to their potential to interact selectively with biological molecules and mimic natural extracellular matrices [19,20].

In this work, we have designed chiral hybrid hydrogels using L/D phenylalanine-based gelators (supramolecular component), PVA, HA, and EGCG. Phenylalanine-based gelators provide a chiral microenvironment and are capable of forming hydrogels by self-assembling in water through non-covalent interactions [21]. These gelators also exhibit good compatibility for co-assembling with various molecules and polymers. PVA contributes to the mechanical strength, adhesive properties, biocompatibility, ease of processing, hydrophilicity, and film-forming capability of the hybrid hydrogels [22,23]. HA is known for its biodegradability, biocompatibility, and remarkable water retention capacity, making it an ideal component for creating a suitable environment for wound healing [24,25]. EGCG, a polyphenol derived from green tea extract, inherently provides antioxidant, anti-inflammatory, and antibacterial properties [26,27,28]. Additionally, it has the ability to co-assemble with supramolecular gelators through non-covalent interactions [29,30]. By integrating these components, we aim to overcome the limitations of previous hybrid hydrogels and create a hybrid hydrogel material with enhanced biological and mechanical properties suitable for advanced biomedical applications.

**Figure 1:**
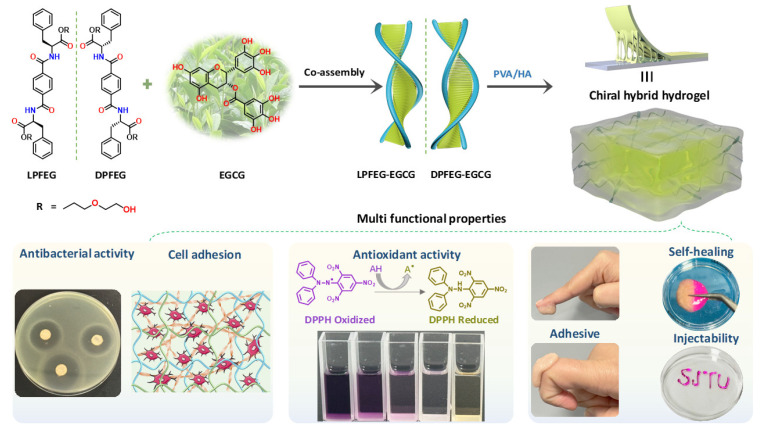
Schematic illustration of preparation of chiral hybrid hydrogels through the co-assembly of EGCG with chiral L/DPFEG assemblies followed by cross-linking with PVA/HA polymers and multifunctional characteristics of prepared chiral hybrid systems.

The following research study focuses on the development of chiral co-assembled hybrid hydrogel systems, designated as LPH-EGCG and DPH-EGCG, which incorporate varying levels of EGCG content. Multi-component co-assemblies in these hybrid gel systems proceeded by the formation of synergetic non-covalent interactions between EGCG and chiral gelator molecules. Subsequently, chirality transfer occurs from these co-assemblies to hyaluronic acid/poly (vinyl alcohol) hydrogel through chain–chain non-covalent interactions (Figure 1). This transfer process could enhance the stability and water-absorbing capacity of the hydrogel and might impart robust mechanical, self-healing, and adhesive properties to the system. In vitro experiments will be performed to demonstrate the expected elevated levels of antibacterial and antioxidant activities as well as enhanced cell migration shown by the hybrid gel system compared to control groups (Figure 1).

## 2. Results and Discussion

### 2.1. Structural and Morphological Characterization

**Figure 2:**
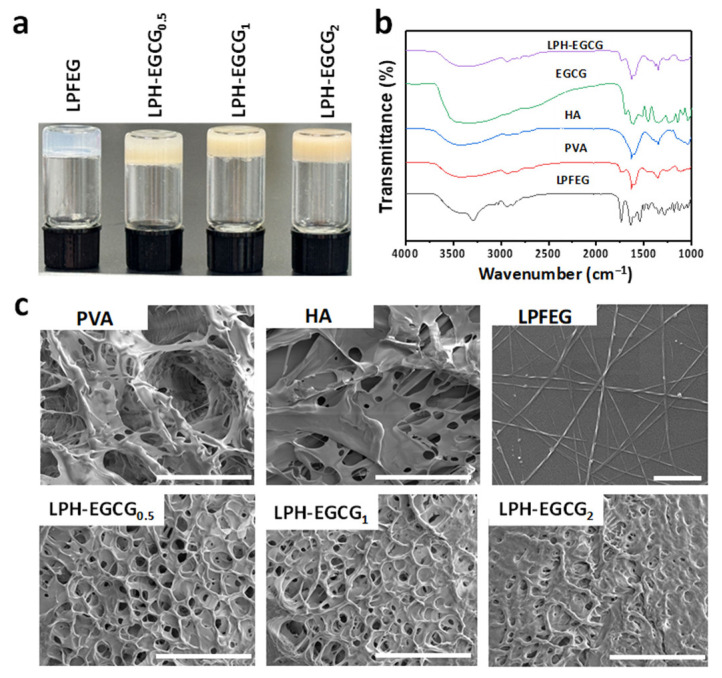
Structural and morphological characteristics of hybrid hydrogels and xerogels. (**a**) Digital photographs presenting the prepared chiral hybrid hydrogels. (**b**) FTIR spectra of pure LPFEG, PVA, HA, LPH-EGCG, and DPH-EGCG co-assembled chiral hybrid xerogels. (**c**) SEM images revealing the microstructure of pure PVA, HA, and chiral co-assembled hybrid xerogel groups (scale bar: 20 μm), and LPFEG hydrogel (scale bar: 3 μm).

LPH-EGCG and DPH-EGCG hybrid gel systems were prepared by mixing fixed contents of L/DPFEG/PVA/HA with varying contents of EGCG through a simple heating-to-cooling method. The prepared hydrogels are presented in Figure 2a. FTIR spectroscopy analyses were carried out for the study of various functional groups present in the components of the prepared hybrid gel systems as well as the possible molecular interactions among them. FTIR experiments were used to investigate the co-assembly and self-assembly mechanism between the components of hybrid hydrogel. Figure 2b presents the FTIR spectra of pristine PVA, HA, and EGCG samples and the xerogels of LPFEG and LPH-EGCG. The FTIR spectrum of pure LPFEG xerogel exhibits characteristic bands at 1640, 1542, and 1736 cm^−1^, which were related to amide I and amide II bending vibrations, and –C=O stretching vibrations [31]. The bands at positions 3291 cm^−1^ and 3451 cm^−1^ represent typical peaks corresponding to N–H bending vibrations in LPFEG xerogel [32]. In the FTIR spectrum of pure PVA, the O–H stretching vibration is observed between 3250 and 3500 cm^−1^, and band broadening is due to both intermolecular and intramolecular hydrogen bonding within the PVA chains. Additionally, characteristic peaks related to C–H stretching vibrations appear at 2925 cm^−1^, while the peak at 1700 cm^−1^ corresponds to the acetyl group (C=O) induced during PVA preparation. A C–H bending vibration related to CH_2_ groups is observed in the range of 1424–1446 cm^−1^ [33]. Moving on to the FTIR spectrum of native HA, characteristic peaks are observed at 2999–3690 cm^−1^ for the –OH and –NH stretching vibrations, associated with hydroxyl and secondary carboxylic acid amide bonds, respectively. A peak around 1620 cm^−1^ is linked to the C–O stretching of carboxylate anion, while symmetric bands related to carboxylate salts are observed at 1357 cm^−1^ [34]. In the spectrum of pure EGCG, prominent peaks are observed at 3365 cm^−1^ and 3357 cm^−1^, corresponding to the O–H group, and 1695 cm^−1^ and 1630 cm^−1^, belonging to the C=O group linking the trihydroxybenzoate group and chroman groups. Peaks at 1351 cm^−1^ and 1243 cm^−1^ are attributed to the O–C=O group, and 1148 cm^−1^ and 1039 cm^−1^ to the O–H groups. The spectrum also depicts the presence of the amide I band at 1691 cm^−1^ and the amide II band at 1548 cm^−1^ [35].

In the FTIR spectrum of the LPH-EGCG hybrid hydrogel, shifts in the frequency of functional characteristic peaks indicate interactions among the components of the system. Notably, the broad band of the phenyl-OH group (∼3689–3018 cm^−1^) suggests interactions among EGCG, LPFEG xerogel, PVA, and HA. The N-H bending vibration-related group in LPFEG xerogel shifts from 3291 cm^−1^ to 3391 cm^−1^, indicating H-bonding interactions in the hybrid gel system. Likewise, the shifting of the C-H bending vibration band associated with CH_2_ groups from 1424–1446 cm^−1^ to 1416–1461 cm^−1^, along with the disappearance of the peak at 1700 cm^−1^ linked to the acetyl group (C=O), signifies the interaction between PVA and other components. The peak around 1620 cm^−1^ linked with C–O stretching of the carboxylate anion in HA shifts towards 1635 cm^−1^, indicating interactions of HA with other components. These findings suggest the presence of non-covalent interactions’ involvement in the co-assembly mechanism.

Morphological analysis was carried out by using SEM to investigate the microscopic structures formed by prepared hybrid hydrogels during the co-assembly process. Figure 2c shows the SEM images of the LPFEG hydrogel and the microstructural features of freeze-dried PVA, HA, LPH-EGCG_0.5_, LPH-EGCG_1_, and LPH-EGCG_2_ co-assembled hydrogels. Upon investigation, the pristine PVA and HA displayed porous morphology. The pure LPFEG hydrogel exhibited three-dimensional helical left-handed fibrous morphology. The SEM images of LPH-EGCG hybrid hydrogels exhibited dual-cross-linked interconnected porous morphology. This cross-linked network structure was created through the co-assembly among PVA, HA, EGCG, and LPFEG. When compared with pure PVA and HA hydrogels, this hybrid system revealed a highly ordered and dense porous structure. Upon comparison of the SEM morphology of three LPH-EGCG hybrid hydrogels, it was observed that with an increase in EGCG content, a denser, more compact, and slightly rough interconnected porous network was observed. This might indicate the greater stability of hybrid hydrogels at higher EGCG contents, which eventually affects the other properties of LPH-EGCH gels in subsequent studies. This is due to the fact that EGCG acted as a cross-linking and strengthening agent and its higher loading led to the formation of a much denser network, suggesting the development of more non-covalent interactions in hybrid systems [36].

**Figure 3:**
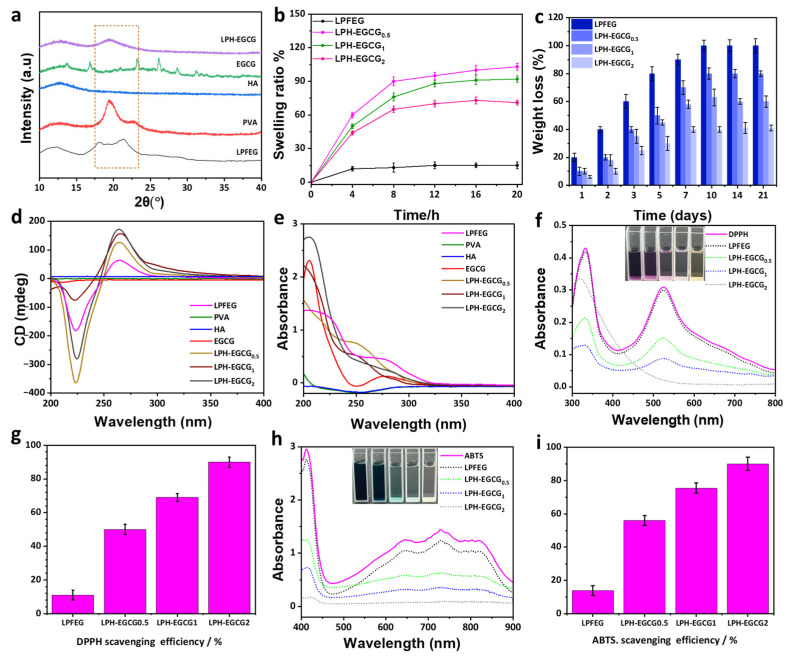
Physical, structural, and optical properties of the hybrid hydrogels. (**a**) XRD diffraction analysis of pristine, PVA, HA, EGCG, and LPFEG xerogels, as well as the co-assembled LPH-EGCG hybrid xerogel. (**b**) Swelling ratio of the prepared LPFEG and LPH-EGCG systems at selected time intervals. (**c**) Weight loss profile of hydrogel samples in PBS on different days. (**d**) CD spectra of neat LPFEG, PVA, HA, EGCG, and LPH-EGCG hybrid chiral gels. (**e**) Their corresponding UV-vis spectra. (**f**) UV-vis spectra present the absorbance of pure DPPH solution and changes in their absorbance by addition of pure LPFEG, and LPH-EGCG hybrid hydrogels, while on top are images showing the change in their colors. (**g**) Scavenging effect of pure LPFEG and chiral hybrid hydrogels evaluated by DPPH-scavenging assays. (**h**) UV-vis spectra exhibiting the absorbance of neat ABTS solution and then changes in its absorbance by addition of different groups including LPFEG and LPH-EGCG gels, while on top are images showing changes in their corresponding solution colors. (**i**) ABTS-scavenging efficiency of chiral hydrogels.

XRD diffraction analyses were carried out in order to determine the mode of the internal packing structure of hybrid hydrogels and possible interactions among the components of the system. Figure 3a shows the XRD patterns of pure LPFEG xerogel, pristine PVA, HA, EGCG, and LPH-EGCG. The XRD diffraction pattern of pure HA exhibited no distinct characteristic diffraction peaks and instead presented a broader pattern, indicating its fully amorphous nature [25]. The XRD pattern of LPFEG xerogel exhibited main characteristic peaks at 2*θ* = 16.95° and 21.30°, which were consistent with previous reports and indicated a lamellar structure [37]. Herein, EGCG, basically a crystalline green tea catechin, displayed multiple characteristic sharp and intense peaks at 2*θ* = 13.67°, 16.82°, 23.18°, 26.14°, 28.59°, and 31.19°, indicating a highly crystalline nature [38]. It has been observed that characteristic peaks of LPFEG xerogel and ECCG disappeared in LPH-EGCG hybrid gel patterns, suggesting the presence of a possible interaction among them.

In pristine PVA hydrogel, distinctive sharp crystalline peaks were observed at 19.20° and 22.91°. This observation aligns with previous research on PVA, acknowledging that its crystalline properties are driven by strong intermolecular interactions among PVA chains via hydrogen bonding [39]. The degree of crystallinity in PVA typically hinges on the arrangement of its chains. However, during the co-assembly process in hybrid hydrogels, the crystallinity of the PVA phase diminishes upon combination with other components. A broadened peak at 2*θ* = 19.43° emerged, while other characteristic peaks of LPFEG xerogel and EGCG disappeared. This indicates a decrease in crystallinity, likely stemming from cross-linking interactions involving EGCG, PVA, and LPFEG, occurring between PVA polymer chains. These interactions lead to the separation of PVA polymer components, disrupting the formation of crystalline structures. The disappearance of the peak at 22.90° in the XRD pattern of LPH-EGCG further supports this trend (Figure 3a). Additionally, inserting polyphenols with HA and LPFEG in PVA also significantly reduces inter- and intramolecular hydrogen bonds in their conjugates. Therefore, crystallinity is decreased and the loose packing structure of the resultant hybrid hydrogel is subsequently increased.

### 2.2. Swelling and Degradation Properties

The water absorption characteristics of prepared chiral hydrogel systems were determined through swelling experiments. The SR of pure LPFEG hydrogel and LPH-EGCG systems is shown in Figure 3b. Observation indicates that morphological features greatly affect the SR of prepared hydrogels, which further significantly impacts their rheological properties. By increasing the soaking time, the swelling of all prepared gels increased rapidly, and hence, they reached maximum swelling within almost 8 h. The SR of pure LPFEG was 20 ±  2.1% lower than hybrid hydrogels with varying EGCG content. The swelling of LPH-ECGC_0.5_ was 103 ± 3.9%, which was slightly higher than LPH-EGCG_1_ (92 ± 2.5%) and LPH-EGCG_2_ (71 ± 1.9%). These findings clearly indicate that by increasing the content of EGCG, the swelling properties decrease gradually, which might be due to the higher hydrophobicity of EGCG. The SR of DPH-EGCG systems is presented in Figure S1a, where DPH-EGCG_0.5_, DPH-EGCG_1_, and DPH-EGCG_2_ exhibited an SR of 95 ± 3.5, 87 ± 1.6, and 70 ± 2.4%, respectively. Moreover, no significant difference was noticed among the SR of LPH-EGCG and DPH-EGCG hybrid gel systems (Figure S1a).

It was found that all hybrid gel systems exhibited increased swelling characteristics compared to pure LPFEG/DPFEG hydrogels. LPFEG and DPFEG demonstrated negligible swelling properties due to their three-dimensional helical nanofibrous network structure which was unable to retain water molecules. Incorporation of PVA, HA, and EGCG in LPFEG/DPFEG resulted in a maximum SR of approximately 103%. This enhancement could be attributed to the incorporation of hydrophilic polymers. PVA and HA, being hydrophilic polymers with porous structural morphology, can retain a large quantity of water molecules. However, it was observed that increasing EGCG content led to a slight decrease in swelling characteristics, although the system still had a high swelling ratio. This phenomenon could be attributed to the more compact, complex, and denser morphology formed at higher concentrations of EGCG due to higher cross-linking density. The structure hinders the formation of a network conducive to water absorption, thereby limiting the interaction of water molecules with the hybrid gel matrix [40].

The in vitro stability profile of prepared LPFEG and hybrid LPH-EGCG hydrogel systems was determined by measuring their corresponding weight loss in phosphate-buffered saline (PBS) (pH 7.4) at 37 °C. Pristine LPFEG hydrogel displayed poor stability and almost disintegrated completely in a physiological environment over a period of 21 days (Figure 3c). However, a relatively lower weight loss (80 ± 3.5%) was noted for LPH-EGCG_0.5_ hybrid hydrogel, whereas LPH-EGCG_1_ and LPH-EGCG_2_ exhibited weight losses of 60 ± 4% and 41 ± 4.1%, respectively, over a span of 21 days. Interestingly, the LPH-EGCG_2_ co-assembled hydrogel was more stable among other groups, implying that EGCG concentration plays a vital role in improving the stability of hybrid hydrogels. In the case of the DPH-EGCG system, a similar trend of stability was observed, where the weight loss profile of DPH-EGCG_2_ was 45 ± 4.3% lower than DPH-EGCG_1_ (68 ± 2.5%) and DPH-EGCG_0.5_ (85 ±  3.5%), indicating higher stability of the co-assembled gel system with higher EGCG content (Figure S1b).

Pure LPFEG gel easily degraded when immersed in PBS solution; however, LPH-EGCG and DPH-EGCG gels with varying contents of EGCG could withstand PBS for longer periods. The introduction of PVA, HA, and EGCG in the hybrid system decreased the rate of degradation and hence increased the stability of the system. For the fabrication, EGCG was added as filler material in the matrix made up of LPFEG/DPFEG, PVA, and HA. EGCG acted as a cross-linking and strengthening material that increased the stability of hydrogels against degradation in PBS solution. This filler material was used previously as a cross-linker in combination with chitosan, cellulose, and other hydrocolloids. Moreover, a gel’s strength/stability highly depends on its network structure. An intertwined gel network is beneficial for producing stronger hydrogels. As shown in SEM images, the addition of higher contents of EGCG made the hybrid gel network denser, rough, and slightly compact, making it highly stable [41].

### 2.3. CD Spectroscopy

Circular dichroism spectroscopy studies provide information related to chirality and are also used to investigate the formation of supramolecular co-assembled nanostructures in chiral hybrid hydrogels. Figure 3d displays the CD spectra of pristine LPFEG hydrogel along with pure PVA, HA, EGCG, and the chiral hybrid LPH-EGCG system with varying contents of EGCG in the range of 200–400 nm. The CD spectrum of pristine LPFEG hydrogel displayed a characteristic positive Cotton effect peak at 263 nm and a negative Cotton effect signal at 225 nm. According to the literature, these characteristic peaks of bare LPFEG gelator were formed by the amide group and 1,4-benzenedicarbonamide chromophore. The positive Cotton effect at 265 nm was owing to the *π–π* intermolecular transition of the central aryl group, while the negative peak at 225 nm was due to the movement of terminal phenyl or amido linkage toward the central aryl group [42]. The pristine PVA, HA, and EGCG did not exhibit any significant signal, which indicates their achiral nature. The supramolecular chiral LPH-EGCG system with varying contents of EGCG exhibited interesting CD signals. In the case of the LPH-EGCG_0.5_ hybrid gel, it showed positive and negative Cotton effect peaks at the same positions as those of pure LPFEG. However, the intensity of the CD peak increases for the chiral hybrid gel system. Similarly, the LPH-EGCG_1_ hybrid hydrogel exhibited the same trend as that of LPFEG assemblies with a more intense signal compared to LPH-EGCG_0.5_ and bare LPFEG. Meanwhile, the hybrid chiral gel system named LPH-EGCG_2_ also exhibited the same position of positive and negative Cotton effect signals with the highest signal intensity compared to the other two hybrid and pure LPFEG assemblies. In UV-vis spectra, the intensities of LPH-EGCG hybrid systems also increase in the same manner. The results indicate that the CD spectra of LPH-EGCG co-assemblies are the same as those of the LPFEG hydrogel without any inversion. However, the signal intensity is enhanced with increased content of EGCG in LPH-EGCG. This suggests the formation of possible stronger H-bonding interactions at higher EGCG loading (Figure 3e). These highly intense negative and positive CD peaks in co-assembled hybrid gel systems might be due to the highly ordered arrangement of intermolecular interactions of amide linkages present within phenylalanine groups and strong H-bonding interactions during co-assembly among components of hydrogels [21].

The CD signal of pure DPFEG hydrogel exhibited a mirror-symmetrical relation to LPFEG hydrogel with a negative Cotton effect peak at 264 nm and a positive effect signal at 223 nm. The spectra of DPH-EGCG chiral hybrid hydrogels exhibited the same trend as DPFEG assemblies with no chiral inversion. Here, the intensity increases like LPH-EGCG with increasing content of EGCG (Figure S2a,b).

### 2.4. Free Radical-Scavenging Properties

Herein, we investigated the free radical-scavenging activity of prepared LPH-EGCG and DPH-EGCG systems at varying EGCG contents in vitro through DPPH and ABTS assays. DPPH is a stable free radical and demonstrates maximum absorption intensity at a wavelength of 517 nm. The analysis of the free radical DPPH assay demonstrated a decrease in absorbance intensity of the DPPH solution upon treatment with the prepared hybrid hydrogels. This change was accompanied by a shift in the dark purple coloration of the pure DPPH solution, indicating the free radical-scavenging capabilities of these prepared hybrid systems (Figure 3f and Figure S3a).

At higher vigorous antioxidant activity, a change in the DPPH solution color was more prominent, along with a significant decrease in UV absorption at 517 nm. Interestingly, it has been observed that the scavenging abilities of both LPH-EGCG and DPH-EGCG hybrid hydrogels for DPPH free radical solutions increase with the concentration of EGCG. The DPPH free radical-scavenging abilities of the prepared LPH-EGCG compound were increased in the following order: LPH-EGCG_2_ > LPH-EGCG_1_ > LPH-EGCG_0.5_. A similar trend was followed by DPH-EGCG hybrid hydrogels at varying EGCG contents (Figure 3g). The DPPH free radical-scavenging efficiency was determined to be 90% for LPH-EGCG_2_, whereas DPH-EGCG_2_ exhibited a DPPH-scavenging efficiency of 84.17% (Figure S3b).

The hybrid gel system displayed a consistent trend of antioxidant activity when subjected to the free radical ABTS+ assay. Notably, as the antioxidant activity increased, the color change in the pure ABTS solution from dark blue/green became more pronounced, accompanied by a decrease in its absorption intensity (Figure 3h and Figure S3c). The pure ABTS solution exhibited a dark blue/green color with maximum absorption intensity at 734 nm. When LPH-EGCG_2_ was employed, the results revealed its superior ability to scavenge ABTS+, reaching approximately 89%, whereas DPH-EGCG_2_ inhibited ABTS+ by around 80% (Figure 3i and Figure S3d). The DPPH- and ABTS-scavenging efficiencies of all prepared hybrid gel systems are reported in Table S1. The findings from the DPPH and ABTS analyses indicate that both LPH-EGCG and DPH-EGCG exhibit remarkable free radical-scavenging abilities, particularly at higher EGCG loadings. This suggests that the antioxidant properties of these prepared systems predominantly arise from the presence of EGCG.

The results obtained from the ROS-scavenging experiments revealed that the antioxidant activity of the hybrid system depends upon the presence of EGCG. EGCG stands out as the most prevalent polyphenol found in green tea with inherent strong antioxidant properties [28]. The mechanism of action of EGCG as a free radical scavenger is primarily linked to its capability for single-electron reduction and its potential to serve as either an electron or hydrogen donor. This means that EGCG molecules react with various free radicals through two mechanisms: they can either carry out hydrogen atom transfer (HAT) reactions, in which the free radical molecules remove a hydrogen atom from the antioxidant molecules (EGCG), and during the second mechanism, the antioxidant (EGCG) itself becomes a free radical and performs the single-electron transfer (SET) reaction. In this reaction, the EGCG molecule provides a single electron to the free radical molecules and becomes a radical cation. When we look into the structure of the EGCG molecule, the ortho-dihydroxyl groups are present on B and D rings, whereas a galloyl moiety is present in three positions. These specific groups enhance the ability of the EGCG molecule to scavenge various types of free radicals including 1,1-diphenyl-3-picrylhydrazyl radicals, 2,2′-azino-bis (3-ethylbenzothiazoline-6-sulfonic acid, superoxide anions, and hydroxyl radicals [26,43,44]. It has been observed that within the hybrid hydrogel system, the antioxidant activity of EGCG remains preserved even upon its encapsulation within the chiral gel system.

### 2.5. Rheological Properties

**Figure 4:**
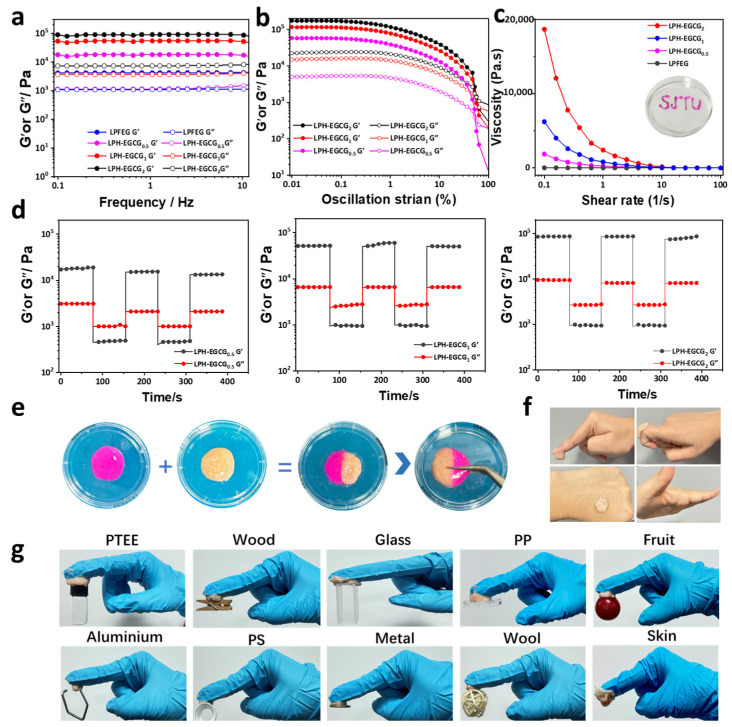
Rheological properties of neat LPFEG and chiral co-assembled hybrid hydrogels. (**a**) Frequency sweep test results at 25 °C. (**b**) Amplitude sweep test measurements of gel systems at room temperature. (**c**) The shear viscosity profile at shear rates ranging from 0.1 to 100/s. (**a**) Pure LPFEG hydrogel and LPH-EGCG hybrid gel system at different concentrations of EGCG. (**d**) The self-healing properties of hybrid hydrogels obtained through a continuous step-strain test at 25 °C and fixed frequency. (**e**) Image exhibiting the macroscopic self-healing property of chiral co-assembled hybrid hydrogel. (**f**) Digital photographs of hybrid hydrogel sticking to human skin at different angles. (**g**) Images displaying sticking of co-assembled hydrogel to various materials.

We carried out a detailed study on the rheology properties of our prepared hybrid hydrogels LPH-EGCG and DPH-EGCG with varying contents of EGCG to investigate their dynamic viscoelastic behavior. During frequency sweep experiments, the storage modulus (*G′*) and loss modulus (*G″*) of hybrid hydrogels were determined as a function of frequency, and all experiments were carried out in room temperature conditions. The results in Figure 4a demonstrate that in all LPH-EGCG hybrid hydrogels at varying EGCG contents, the storage modulus is greater than the loss modulus over the entire range of frequency. This indicates that all hydrogels were capable of maintaining a suitable elastic state. It was noticed that with an increase in frequency, the storage and loss moduli changed with a little amplitude, indicating weak frequency dependence; therefore, all prepared LPH-EGCG hydrogels were capable of having good tolerance to external forces [45,46]. These findings suggest that the LPH-EGCG hybrid systems exhibited good mechanical performance. The pure LPFEG and LPH-EGCG_2_ revealed a *G′* of 4208 Pa and 85,127 Pa, respectively. The significant improvement in the rheological properties of hybrid hydrogels compared with the pristine LPFEG hydrogel was mainly attributed to the network cross-linked structure formed by stronger non-covalent interactions among the PVA, HA, EGCG, and LPFEG during co-assembly, as indicated by SEM analysis. Furthermore, we also investigated the rheological performance of three prepared hybrid gel systems with varying EGCG contents. A significant increase in *G′* values was observed by increasing the EGCG content. Average *G′* values ranged from 17,025 to 85,127 Pa with an increase in the content of EGCG from 0.5 to 2mg in hybrid co-assembled systems. This increase in *G′* values demonstrates that higher cross-linking agent (EGCG) content can enhance non-covalent interactions, therefore providing good tolerance to external applied forces and leading to easy energy storage and dissipation throughout the hybrid system. As revealed by SEM analysis, higher EGCG content resulted in a denser and more compact network responsible for the enhancement of rheological properties [47].

The DPH-EGCG hybrid hydrogels at varying EGCG contents demonstrated weak frequency dependence behavior. However, the storage modulus was higher than the loss modulus for three DPH-EGCG systems. The storage modulus of the DPH-EGCG_2_ hybrid is 63,579 Pa and is slightly lower than LPH-EGCG_2_ gel (Figure S4a). These findings indicate that the L-enantiomer-based hybrid gel system (LPH-EGCG) is more effective and forms stronger gels compared with D-enantiomeric systems (DPH-EGCG). The better mechanical behavior of LPH-EGCG gel might be due to the difference in chirality that further impacts the organization at the micro level (fibers) and macroscopic level (hybrid hydrogel). The frequency sweep experimental findings revealed that the chiral stereochemistry may have a little influence on the rheological properties.

The oscillatory amplitude sweep experiment was performed at a fixed frequency value of 1 Hz at room temperature. Experimental findings indicate that both hybrid hydrogel systems LPH-EGCG and DPH-EGCG displayed linear viscoelastic behavior where the storage and loss moduli were almost independent of applied strain. It was also observed that the storage and loss moduli increased by increasing the content of EGCG. Moreover, values of *G*′ were higher than *G*′′, and there was a point where storage and loss moduli crossed over one another, therefore presenting a shift from “solid-like” to “fluid-like” behavior (Figure 4b and Figure S4b). These outcomes suggest the dominant elastic behavior of hydrogels [48].

As shown in Figure 4c and Figure S5, LPH-EGCG hybrid systems exhibited high viscosity values and increases at higher EGCG contents. With an increase in shear rate, the viscosity of the three prepared systems decreases, revealing the shear-thinning behavior. The viscosity profile showed the significant injectability properties of LPH-EGCG. Similar findings were obtained for three prepared DPH-EGCG hybrid systems.

To evaluate the self-healing properties of LPH-EGCG hybrid co-assembled hydrogels at varying EGCG contents, a time sweep experiment was carried out at room temperature by varying the strain from 0.2% (low) to 100% (high) at the same frequency of 10 Hz. This experiment revealed the shear recovery and rapid shear-thinning behavior of prepared gel systems. Initially, these hydrogels were kept at a constant low strain value (0.2%) where the storage modulus was higher than the loss modulus. Under high strain (0.2–100%), a rapid inversion of storage modulus and loss modulus was observed, where *G′* of the hybrid hydrogel system decreased and became lower than *G″*, indicating shear-thinning behavior. At this stage, LPH-EGCG was transferred from the gel to sol phase; in other words, this step also indicates the complete breakdown of the hybrid system. When high strain (100%) was finally decreased to low strain (0.2%), the storage modulus of LPH-EGCG was recovered rapidly, indicating that all hydrogels have recovery properties. The self-recovery behavior was continued for numerous cycles, and the outcomes indicate the excellent shear recovery behavior of hybrid hydrogels (Figure 4d). The time sweep experiment of DPH-EGCG hybrid hydrogels displayed self-healing behavior in DPH-EGCG_0.5_, DPH-EGCG_1_ and DPH-EGCG_2_ (Figure S5) [49].

Self-healing results were further confirmed through macroscopic recovery experiments. As shown in Figure 4e, a small piece of LPH-EGCG was dyed with rhodamine B and placed with an unstained piece. After 8 h, it was observed that the two pieces of hybrid hydrogel gradually reattached together and did not fall apart easily, indicating a much stronger self-healing ability. The self-healing results demonstrated that a dynamic physical cross-linked network structure was created in these gels through the development of non-covalent interactions between fibers of LPFEG and EGCG, PVA, and HA polymer chains. This network system contributed to energy dissipation in prepared hydrogels that allowed the LPH-EGCG to be dynamically remodeled upon rupture [50].

We analyzed the adhesion of the prepared hybrid system to human skin, as shown in Figure 4f. The developed hybrid dressing was able to attach completely to human skin. Moreover, bending at different positions did not cause detachment from the skin, indicating strong adhesion to human skin. These adhesive features make the LPH-EGCG hybrid a promising dressing material to apply to the skin surface. In addition to this, LPH-EGCG showed excellent adhesion to a variety of materials with different load capacities, such as polytetrafluoroethylene (PTFE), wood, glass, polypropylene (PP), fruit, aluminum, polystyrene (PS), metal, wool, and chicken skin (Figure 4g).

### 2.6. Antibacterial Properties

**Figure 5:**
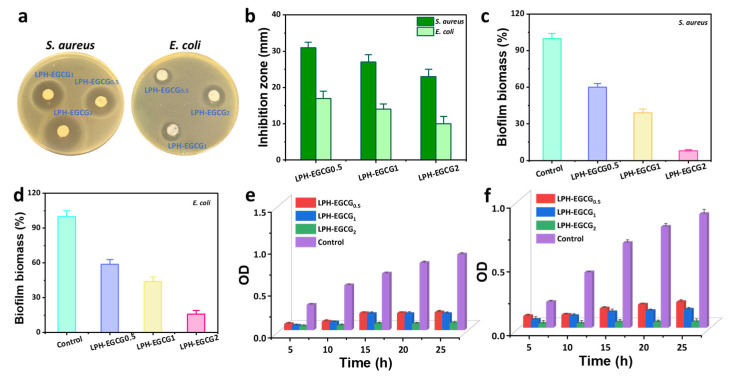
Antibacterial study of hybrid hydrogels. (**a**) Digital photos presenting inhibition zone formed by LPH-EGCG hybrid gel against *S. aureus* and *E. coli*. (**b**) Corresponding quantitative measurements of diameter of zone of inhibition. (**c**,**d**) Biofilm destruction and corresponding measurement of biofilm biomass caused by LPH-EGCG chiral co-assembled hybrid hydrogels at varying loadings of EGCG. Time-dependent antibacterial activity measurement of chiral co-assembled hybrid hydrogels LPH-EGCG and DPH-EGCG against (**e**) *S. aureus* and (**f**) *E. coli* at OD 600 nm.

The Gram-positive bacterial strain *S. aureus* and the Gram-negative *E. coli* are the most common types of pathogens that cause major infections in human bodies especially in infected wound areas [51]. Therefore, these two bacterial groups were selected to evaluate the in vitro antibacterial activity of LPH-EGCG and DPH-EGCG co-assembled hydrogels with varying EGCG contents through an agar disk diffusion process. As shown in Figure 5a, prominent inhibition zones were observed after treatment with LPH-EGCG hydrogels. When the content of EGCG in hybrid hydrogels was increased, the diameters of zones of inhibition also increased, indicating that the antibacterial activity of the system was mainly due to the presence of EGCG (Figure 5b). Table S2 presents quantitative measurements of the diameter of zones of inhibition created by LPH-EGCG and DPH-EGCG hydrogels. Experimental outcomes revealed a larger-diameter zone of inhibition when the hybrid gel system was treated with the bacterial strain *S. aureus*, revealing greater antibacterial activity against Gram-positive bacteria. However, we also observed that LPH-EGCG exhibited slightly higher antibacterial activity than DPH-EGCG at all concentrations of EGCG (Figure S6a,b).

The influence of LPH-EGCG and DPH-EGCG hybrid gel systems on prepared biofilms was studied through a crystal violet assay (where OD values were determined at 570 nm). The destruction of prepared biofilms caused by LPH-EGCG and DPH-EGCG with varying concentrations of EGCG was investigated. The prepared *S. aureus* and *E. coli* biofilms stained by crystal violet gradually disassembled when treated with LPH-EGCG and DPH-EGCG hybrid systems at increased content of EGCG, as shown in Figure S7a. These outcomes indicate that the prepared biofilms were significantly damaged by LPH-EGCG and DPH-EGCG hybrid hydrogels. The quantitative measurements of biofilm mass of *E. coli* and *S. aureus* after treatment with LPH-EGCG and DPH-EGCG hybrid are presented in Figure 5c,d and Figure S8a,b. The experimental results indicate that the co-assembled hybrid system can destroy the prepared biofilms. Moreover, the antibacterial activity of LPH-EGCG systems was slightly higher than DPH-EGCG gels (Table S3). With increased content of EGCG in the hybrid system, more destruction of biofilms was observed. These experimental results proved the good antibacterial effect of hybrid hydrogels on prepared bacterial biofilms.

To further study the antibacterial properties of hybrid co-assembled gels, they were transferred into 24-well plates. Subsequently, bacterial solution was added, followed by incubation in a mechanical shaker (90 rpm) at 37 °C for time intervals of 5 h, 10 h, 15 h, 20 h, and 25 h. The growth of bacteria was assessed by measuring the optical density at 600 nm (OD_600_) using a synergy H1 multi-mode microplate reader. The outcomes were consistent with the results obtained from the above-mentioned agar well diffusion method. Thus, the data depicted in Figure 5e,f and Figure S8 demonstrated the EGCG concentration-dependent inhibitory effect of LPH-EGCG and DPH-EGCG hybrid hydrogels on the tested bacterial strains at specified time intervals.

**Figure 6:**
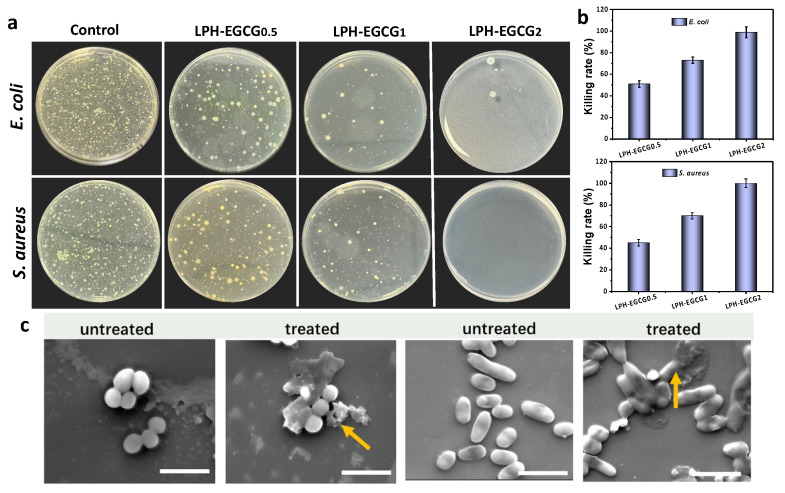
Antibacterial study of hybrid hydrogels. (**a**) Photographs of bacterial colonies formed by *S. aureus* and *E. coli* bacteria when treated with LPH-EGCG hybrid gel systems with different contents of EGCG. (**b**) Corresponding rate of bacterial inhibition. (**c**) SEM images exhibiting the morphology of bacterial cells (*S. aureus* and *E. coli*) before and after 5 h treatment with LPH-EGCG (scale bar: 3 μm). The arrows in the figure indicates the damaged bacterial cells.

Through in vitro antibacterial assays, the antibacterial activities of prepared co-assembled hybrid hydrogels named LPH-EGCG and DPH-EGCG at different EGCG contents were verified through a CFU plate-counting method. As shown in Figure 6a and Figure S9a, both hybrid hydrogel groups displayed excellent antibacterial activity against *S. aureus* and *E. coli* as compared to the blank group. With an increase in the EGCG content in both LPH-EGCG and DPH-EGCG hydrogels, a reduction in the number of bacterial colonies was observed. The calculated inhibition rates for LPH-EGCG_0.5_, LPH-EGCG_1_, LPH-EGCG_2_, DPH-EGCG_0.5_, DPH-EGCG_1_, and DPH-EGCG_2_ are presented in Table S4. LPH-EGCG and DPH-EGCG displayed a more than 90% inhibition rate against *S. aureus* and more than 80% against *E. coli* upon loading of EGCG at 2 wt % (Figure 6b and Figure S9b). The results obtained from the CFU colony count method are in complete agreement with the agar well diffusion method and time-dependent antibacterial activity analysis. The findings from all reported methods revealed the EGCG content-dependent antibacterial activity of the developed gels.

Although the prepared hybrid gel systems revealed significant antibacterial performance, the underlying antibacterial activity mechanism has yet to be discussed properly. In this study, we prepared a co-assembled hybrid hydrogel by combining L and D phenylalanine-based hydrogels with PVA, HA, and EGCG. According to the literature, pure LPFEG, DPFEG, PVA, and HA do not exhibit any prominent antibacterial activities. However, the catechin (EGCG) belongs to a family of polyphenols possessing excellent antibacterial activities against various kinds of pathogenic bacteria. Thus, the antibacterial activity of the system solely depends upon the presence and concentration of EGCG, whose inherent antibacterial efficacy remained intact upon encapsulation within the gel system. The primary antibacterial mechanism involves the cooperativity of LPH-EGCG and DPH-EGCG chiral hybrid structures. This is attributed to the development of effective non-covalent interactions among the components of the hybrid system, and the bioactivities of EGCG released from these hybrid hydrogels are further enhanced by these chiral structures [18]. The already reported mechanisms of antibacterial activity exhibited by EGCG against both Gram-negative and Gram-positive bacteria can be categorized as follows: (1) disruption of bacterial cell membranes and walls, (2) inhibition of virulence factors such as extracellular matrix components, (3) inhibition of various intracellular enzymes, (4) induction of oxidative stress, (5) damage to bacterial DNA, and (6) chelation of iron [52]. However, this study observed that damaged bacterial cells treated by hybrid hydrogels exhibited membrane destruction and leakage of cellular components. The changes in the morphologies of *S. aureus*, and *E. coli* bacterial cells after 5 h treatment with hybrid hydrogel (LPH-EGCG and DPH-EGCG) are presented in Figure 6c and S10, where most of the cells were misshapen and disintegrated. Observations from SEM investigations suggest that the general antibacterial mode of action demonstrated by hybrid systems is associated with the destruction of bacterial cell membranes. This involves EGCG binding to the bacterial cell membrane, causing damage to its morphology. The presence of EGCG in hybrid hydrogels is also speculated to affect bacterial membrane permeabilization, resulting in the disruption of membrane integrity and leakage of intracellular materials such as proteins and nucleotides. Consequently, cell membrane shrinkage and morphological changes occur, ultimately causing cell death. This unique antibacterial mechanism of membrane destruction allowed the hybrid gel system to display broad-spectrum antibacterial performance.

### 2.7. Cell Study

**Figure 7:**
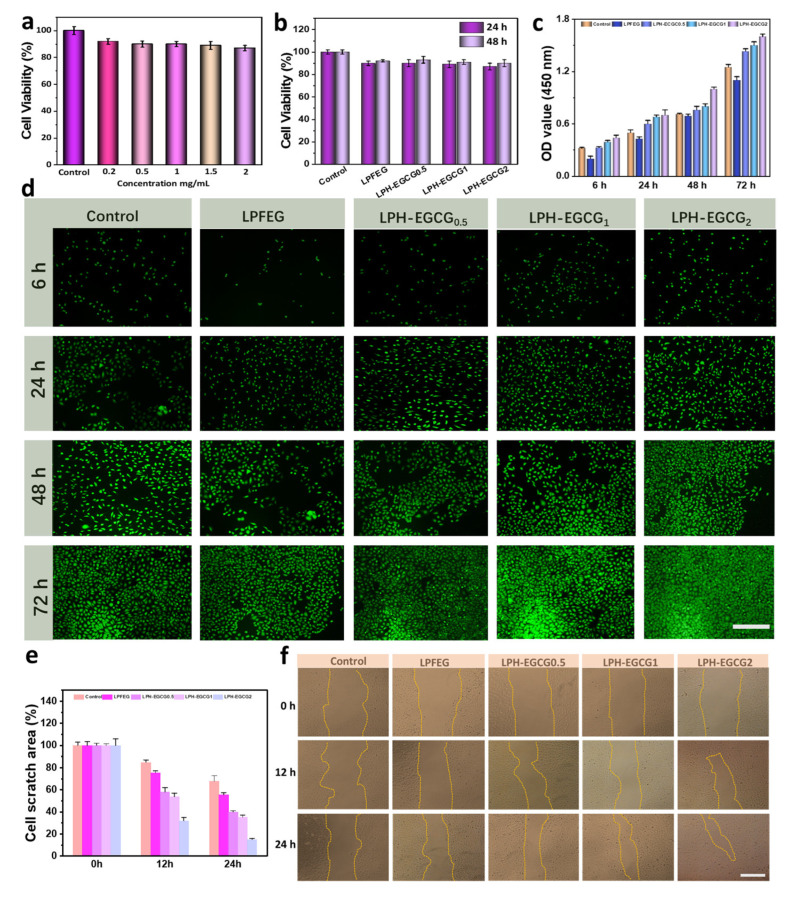
Cell viability study of (**a**) various concentrations of EGCG when incubated for 24 h with HUVEC cells. (**b**) LPFEG and LPH-EGCG chiral hybrid systems co-cultured with HUVEC cells for 24 and 48 h. (**c**) Quantitative measurements of cell adhesion of HUVEC cells when co-cultured with LPFEG and LPH-EGCG chiral hybrid systems for 6 h, 24 h, 48 h and 72 h incubation time periods. (**d**) Fluorescence microscope images of live and dead HUVEC cells after being cultured for different incubation time periods with control (PS), LPFEG, and various chiral hybrid LPH-EGCG systems (scale bars: 100 μm). (**e**) Quantitative measurements of relative cell scratch area measured in control and chiral hybrid groups after 0, 12, and 24 h incubation times. (**f**) Images of HUVEC cell scratch areas observed at specific time intervals, when treated with various groups (scale bars: 50 μm).

To explore the biocompatibility of the chiral hybrid gel system against HUVEC cells, the MTT assay was utilized. Figure 7a shows that the cell viabilities at various concentrations of EGCG are above 80%, indicating its non-toxicity. Moreover, the cell viability profile of the LPH-EGCG and DPH-EGCG chiral hybrid systems revealed good biocompatibility when cultured with HUVEC cells for 24 h and 48 h (Figure 7b and Figure S11). It is worth mentioning here that no significant difference was observed in the biocompatibilities of the LPH-EGCG and DPH-EGCG chiral hybrid systems, and HUVEC cells can potentially grow in medium containing these chiral gels.

In order to further investigate the influence of chiral hybrid gels in promoting cell adhesion and proliferation, HUVEC cells were seeded on chiral LPH-EGCG and DPH-EGCG hybrid gel films, while PS was used as a control. The cells were co-cultured with chiral hybrid gel films for different incubation periods of 6, 24, 48 and 72 h in an incubator at 37 °C and 5% CO_2_. It was observed that during the 6 h incubation period, the number of living cells attached to the LPH-EGCG hybrid gel films at all concentrations of EGCG was higher compared to those adhered to chiral DPH-EGCG hybrid gel films and PS. The LPH-EGCG_2_ exhibited the highest cell count adhered on its surface. The same trend was noted throughout all incubation periods (Figure 7c). It is noteworthy that the smaller number of living cells attaching to chiral DPH-EGCG at different EGCG concentrations compared to LPH-EGCG chiral systems was not attributable to cell mortality, as confirmed by cytotoxicity studies of the DPH-EGCG hybrid system (Figure S12). These chiral systems exhibited a cell viability of 80%. This difference in cell adhesion might be due to the chirality of LPFEG and DPFEG hydrogels, suggesting that left-handed chiral fibers in LPH-EGCG hydrogel provide a type of chiral microenvironment that facilitates cell adhesion and further proliferation [18].

Furthermore, live/dead staining assay images depict that cells attached to the surface of chiral hybrid films form long filopodia and are in a healthy state. With an increase in the incubation period, the number of metabolically active cells increased, and after 72 h of incubation, the surface of LPH-EGCG_2_ was completely covered with living cells. Furthermore, it was observed that in both LPH-EGCG and DPH-EGCG chiral hybrid systems, an increase in the EGCG content led to enhanced cell adhesion, and both hybrid systems exhibited greater cell proliferation compared to pristine LPFEG and DPFEG hydrogels (Figure 7d and Figure S13). This might be due to the increase in stiffness of hybrid gel systems at higher content of EGCG.

Cell proliferation was further confirmed through cell migration studies. The cell scratch assay depicted a gradual diminishment in the cell scratch area over time. Compared to the control and pure LPFEG and DPFEG hydrogels, co-incubation with chiral LPH-EGCG and DPH-EGCG hybrid hydrogel formulations resulted in a more accelerated decrease in the scratch site, therefore facilitating quicker HUVEC cell migration (Figure 7e and Figure S14). It was observed that chiral LPH-EGCG hybrid gel systems allowed a greater decrease in scratch area compared to DPH-EGCG chiral gels, and with an increase in EGCG content in hybrid gel systems, a greater reduction in scratch area was observed. In particular, after 24 h of incubation, the LPH-EGCG_2_ hybrid hydrogel displayed the highest migration rate across all groups, where only 10.1% of the region was not covered with living cells (Figure 7f and Figure S15). These findings consistently align with earlier observations on cell adhesion.

## 3. Conclusions

The development of multifunctional biomaterials typically requires a complex synthesis process that combines biologically active molecules with hydrogel materials. However, in our research, we employed a simpler co-assembly method to create hybrid gel systems with diverse functionalities within a single biological structure. A significant advancement in our approach was the introduction of chiral properties, which meticulously controlled the cell adhesion and migration characteristics of the hydrogels. We fabricated chiral co-assembled hybrid hydrogels, named LPH-EGCG and DPH-EGCG, using this straightforward preparation technique. The creation of multi-component co-assemblies within these gel matrices was driven by synergistic non-covalent interactions between EGCG and L/DPFEG chiral gelator molecules. Further cross-linking with PVA/HA not only improved the hydrogels’ stability and water absorption but also enhanced their mechanical strength, self-healing, adhesiveness, and injectability. In vitro experiments demonstrated that the hybrid gel systems exhibited significant antibacterial and antioxidant activities, outperforming control groups due to the presence of EGCG. Additionally, CCK-8 and live/dead assays revealed that LPH-EGCG and DPH-EGCG chiral assemblies had distinct effects on the adhesion and growth of HUVEC cells. Notably, LPH-EGCG hydrogels created a chiral microenvironment that facilitated cell growth and migration. These findings suggest that the chiral LPH-EGCG hybrid system, with its multiple functionalities, holds great potential for various tissue engineering applications.

## 4. Materials and Methods

### 4.1. Experimental Chemicals and Reagents

All the chemical compounds and solvents required for this specific study are mentioned in the Supporting Information (SI).

### 4.2. Synthesis of Chiral L-Phenylalanine- and D-Phenylalanine-Based Gelators (LPFEG and DPFEG)

The LPFEG and DPFEG gelators were synthesized using the standard reported procedure, and the complete synthesis method is mentioned in the SI.

### 4.3. Preparation of Chiral Co-Assembled Hybrid Hydrogels

The chiral co-assembled LPH-EGCG and DPH-EGCG hybrid hydrogel systems were prepared through a simple heating-to-cooling approach. First of all, a stock solution made up of 5 wt % (50 mg) PVA and 1 wt % (10 mg) of HA was prepared by dissolving corresponding amounts of PVA and HA powder in DI water under vigorous stirring and heating. After degassing by sonication for 1 h, a clear solution was obtained. Then, in 1 mL DI water, 3.0 mg of LPFEG/DPFEG gelator and corresponding EGCG at different contents were added, and this suspension was heated at 80 °C until a homogeneous solution was obtained. Finally, this suspension was mixed with PVA/HA solution, and the resultant solution was heated at 80 °C for 30 min and allowed to rest and cool down at room temperature for a while until the gel solidified completely. Finally, the formation of co-assembled hybrid hydrogel was confirmed through a vial inversion method. The pure LPFEG and DPFEG assemblies at a gelator concentration of 3 mg/mL were also prepared through the same heating-to-cooling approach for comparison. The specific amounts of components for the preparation of hydrogels are mentioned in Tables S1 and S2 in the Supporting Information.

### 4.4. Structural Characterizations of Chiral Co-Assembled Hybrid Hydrogels

The microstructures of prepared hybrid xerogels were examined through a Raman image-scanning electron microscope (instrument model: RISE MAGNA, manufactured by TESCAN Czech Republic). For SEM sample preparation, hydrogel samples were first diluted and then freeze-dried to obtain xerogels. The intermolecular interactions among the components of the hybrid gel system were determined through a Perkin Elmer spectrum 100 FTIR spectrometer in the wavenumber range of 400–4000 cm^−1^. A D8 Advance X-ray Diffractometer (manufactured by Bruker corporation, Karlsruhe, Germany) was used to detect the crystal structure of lyophilized hydrogel samples. Furthermore, circular dichroism (CD) and corresponding UV-vis curves of prepared hybrid hydrogels were recorded with a JASCO J-815 spectrophotometer at room temperature in the wavelength range of 200–600 nm in order to obtain further insight into the structure and chirality of hybrid gel systems.

### 4.5. Physical Characterization of Chiral Co-Assembled Hybrid Hydrogels

To assess the swelling characteristics of the synthesized hydrogels, each freeze-dried sample was weighed (*W_i_*) and immersed in PBS solution at pH 7.4 and 37 °C. Following a predetermined interval, excess moisture on the sample surface was blotted dry with filter paper, and the sample was reweighed (*W_f_*). The swelling ratio of hydrogels was calculated following Equation (1).

(1) $$\mathrm{Swelling}\;\mathrm{ratio}=\frac{W_{f}}{W_{i}}\times 100\%$$

To observe degradation, prepared hydrogels were weighed as *W_day_*_0_, then immersed in PBS at 37 °C. At intervals, the hydrogels were dried with filter paper and weighed as *W_dayN_*. The degradation ratio was calculated by the formula mentioned in Equation (2) [5].

(2) $$\mathrm{Degradation}\;\mathrm{ratio}=\left[\frac{{(W}_{day0}-W_{dayN})}{W_{day0}}\right]\times 100\%$$

### 4.6. Rheological Properties, Macroscopic Self-Healing, and Adhesive Test of Chiral Co-Assembled Hybrid Hydrogels

The rheological test was conducted using an AR G2 rheometer. The prepared hybrid gels were placed on 20 mm diameter parallel plates with a 1 mm fixed gap. Two different studies were conducted: the first was a dynamic frequency sweep test at a constant sinusoidal shear strain of 0.5% with varying frequency from 0.1 to 100 Hz, and the second was a strain amplitude sweep test at a constant frequency of 1.0 Hz with varying strain from 0.01 to 100, at room temperature. The shear rate was also increased from 0.01 to 100 s^−1^ linearly in order to determine the shear viscosity of the prepared hybrid hydrogel system. In addition, different matrix materials were used in order to determine the macroscopic adhesion properties of the prepared hybrid gel system.

Self-healing properties were observed when a freshly prepared hybrid gel was cut down into two pieces of equal size, out of which one piece was dyed with rhodamine B. Then, the two hybrid gel pieces were placed together again, and the self-healing characteristic of the hybrid hydrogel was noticed after 8 h.

### 4.7. Free Radical-Scavenging Properties of Chiral Co-Assembled Hybrid Hydrogels

The stable radical DPPH (DPPH·) and cation radical ABTS (ABTS·+) were used for the study of free radical-scavenging properties of chiral co-assembled hybrid hydrogels. The free radical-scavenging properties of pure LPFEG hydrogel and LPH-EGCG and DPH-EGCG hybrids with varying EGCG contents were determined following the method reported in the SI.

### 4.8. In Vitro Antibacterial Properties of Chiral Co-Assembled Hybrid Hydrogels

To assess the antibacterial efficacy of the synthesized co-assembled chiral hydrogels, Gram-negative bacteria (*E. coli* DH10B) and Gram-positive bacteria (*S. aureus* Mu50) were utilized. Antibacterial activity was first analyzed through the formation of inhibition zones. Briefly, 10 mL of freshly prepared bacterial culture medium with bacterial strain solution was poured into individual sterilized Petri dishes. Then, all the hybrid hydrogel samples with varying EGCG contents were cut down into circular disks (6 mm) and placed on the surface of agar plates. After 24 h of incubation, the corresponding diameters of the zones of inhibition formed by co-assembled hybrid gels were measured. In addition to this, a time-dependent antibacterial activity experiment was also carried out, in which 24-well plates were used. Initially, the wells were filled with LPH-EGCG and DPH-EGCG hybrid hydrogels containing different amounts of EGCG. Next, 1 mL of bacterial solution was added to the 24-well plates containing the hybrid hydrogel samples. These plates were then placed in a mechanical shaker set at 90 rpm and maintained at 37 °C for varying time intervals, including 5 h, 10 h, 15 h, 20 h, and 25 h. At each designated time interval, the microbial growth was accessed by measuring the OD values through a multi-mode microplate reader

The antibacterial efficacy of the synthesized co-assembled hybrid hydrogels was also evaluated by determining the inhibition rate using the spread plate or colony-forming unit (CFU) counting method against *S. aureus* and *E. coli* bacterial strains. A bacterial solution with 1 × 10^6^ CFUs/ml was co-cultured with hybrid gel and subsequently incubated at 37 °C for a duration of 5 h. Following this incubation period, the co-culture solution was diluted, and 100 μL of the diluted solution was inoculated onto an LB agar plate, which was then incubated at 37 °C. After a 24 h incubation period, the number of CFUs was determined. This experimental procedure was repeated three times for each experimental group containing hybrid gel samples. A control group, comprising bacterial suspension without the presence of a hydrogel sample, was also included for comparison. The inhibition rate (*HR*) of LPH-EGCG and DPH-EGCG hybrids with varying EGCG contents was calculated using the following formula:

(3) $$\mathrm{HR}=\left[\frac{\left(A_{control}-A_{sample}\right)}{A_{control}}\right]\times 100\%$$

where $A_{control\;}$ and $A_{sample}$ represent CFUs in the control and hybrid hydrogel groups, respectively.

### 4.9. In Vitro Biofilm Destruction by Chiral Co-Assembled Hybrid Hydrogels

Bacterial strain suspensions including *S. aureus* and *E. coli* were diluted with tryptone soy broth (TSB) medium and dispensed into separate wells of 96-well plates (100 µL per well). These plates were then incubated at 37 °C without disturbance for a duration of 72 h. To maintain optimal growth conditions, the TSB medium was refreshed every 24 h. Following the incubation period, the formed biofilms adhering to the bottom of the wells became visible upon discarding the supernatant. The resulting biofilms were gently washed three times with PBS solution. Subsequently, the biofilms were subjected to treatment with control (PBS) and LPH-ECGC and DPH-EGCG hybrid hydrogel samples with varying EGCG contents. The residual biofilms were stained with crystal violet (CV) solution for approximately 15–20 min, followed by rinsing with PBS and air drying at room temperature. The bound CV was then solubilized in 95% ethanol, and the extent of bacterial biofilm destruction was quantified by measuring the optical density (OD) values at 570 nm using a microplate reader. All experiments were conducted in triplicate to ensure statistical robustness.

### 4.10. Mechanism of Antibacterial Activity by Chiral Co-Assembled Hybrid Hydrogels

Scanning electron microscopy was employed to analyze the morphological changes of bacterial cells before and after treatment with co-assembled hybrid hydrogels. Initially, bacterial solutions were deposited onto silicon wafers, both with and without treatment with chiral hybrid hydrogel specimens. Subsequent to this, 1 or 2 drops of 2% glutaraldehyde solution were added to facilitate the fixation of bacterial cells. Following this, dehydration of bacterial cells was conducted using a series of ethanol solutions (50%, 70%, 90%, 100%). Lastly, the silicon wafers containing the bacterial cells were subjected to nitrogen drying to ensure the preservation of cellular morphology [53].

### 4.11. Biocompatibility and Cell Adhesion Study of Chiral Co-Assembled Hybrid Hydrogels

The biocompatibility, cell adhesion, and proliferation characteristics of hybrid co-assembled LPH-EGCG and DPH-EGCG hydrogels were investigated using HUVEC cells. Thin films of all developed hydrogels were prepared at the bottom of 96-well plates. Subsequently, 100 μL of cell solution was seeded into each well. After approximately 6 h, the solution from each well was aspirated, followed by three washes with PBS. Then, 100 μL of fresh DMEM solution was added to each well, and the plates were incubated at 37 °C. Cell adhesion and proliferation were assessed at intervals of 6 h, 24 h, 48 h, and 72 h of incubation. Furthermore, the quantification of cell viability and proliferation was conducted using the cell counting kit 8 (CCK-8) assay, which utilizes a water-soluble tetrazolium salt. For the quantification analysis, 10 μL of CCK-8 solution was added to the 96-well plates containing cells with hydrogels, and cell viability was determined. Following the respective incubation periods (6 h, 24 h, 48 h, and 72 h), the HUVEC cells were rinsed with PBS solution (pH = 7.4) and live/dead fluorescence assays were carried out. The cells were mixed with a staining mixture composed of calcein and propidium iodide (PI) (PBS) solution. Following an incubation period of approximately 20–30 min, confocal laser scanning microscopy was used to analyze the live/dead cell images.

### 4.12. Cell Migration Studies of Chiral Co-Assembled Hybrid Hydrogels

In the cell scratch test assay, the hybrid co-assembled hydrogels were introduced into 24-well cell culture plates containing HUVEC cells. Following a 24 h incubation period, the initial positions of the cells were marked by creating a straight line using a 200 μL yellow pipette tip. Subsequently, the cells were washed twice with PBS to remove any suspended cells and cellular debris. Following the washes, 1 mL of culture medium was added to each well, and each sample was tested in triplicate. The cultures were then placed in a humidified environment at 37 °C with 5% CO_2_. After 12 and 24 h of incubation, the cells were photographed under a microscope, and the scratch areas were quantitatively analyzed using Image J software (version 1.8.0).

## Data Availability

The original contributions presented in the study are included in the article/Supplementary Materials, further inquiries can be directed to the corresponding author.

## Supplementary Materials

The following supporting information can be downloaded at: https://www.mdpi.com/article/10.3390/gels10080489/s1, Figure S1: (a) Swelling ratio of prepared DPFEG and DPH-EGCG systems at selected time interval. (b) Weight loss profile of hydrogel samples in PBS at different days; Figure S2: (a) CD spectra of neat DPFEG, PVA, HA, EGCG and DPH-EGCG hybrid chiral gels. (b) Showed their corresponding UV-vis spectra; Figure S3: (a) UV-vis spectra present the absorbance of pure DPPH solution and change in their absorbance by addition of pure DPFEG, and DPH-EGCG hybrid hydrogels, while on top are images inserted showing change in their colors. (b) Scavenging effect of pure DPFEG and chiral hybrid hydrogels evaluated by DPPH scavenging assays (c) UV-vis spectra exhibiting the absorbance of neat ABTS solution and then change in its absorbance by addition of different groups naming DPFEG, and DPH-EGCG gels, while on top are images showing change in their corresponding solution colors. (d) ABTS scavenging efficiency of chiral hydrogels; Figure S4: Rheological properties of neat DPFEG and chiral co-assembled hybrid hydrogels. (a) Frequency sweep test results at 25 °C. (b) Amplitude sweeps test measurements of gel systems at room temperature. (c) The shear viscosity profile at shear rate ranging from 0.1 to 100/s (a) pure DPFEG hydrogel and DPH-EGCG hybrid gel system at different concentration of EGCG; Figure S5: The self-healing properties of hybrid hydrogels obtained through continuous step-strain test at 25 °C and fixed frequency; Figure S6: (a) Digital photos presenting the inhibition zone formed by DPH-EGCG hybrid gel against *S. aureus* and *E. coli*. (b) Corresponding quantitative measurements of diameter of zone of inhibition; Figure S7: (a) Biofilm destruction and (b) and (c) corresponding measurement of biofilms biomass caused by DPH-EGCG chiral co-assembled hybrid hydrogels at varying loading of EGCG; Figure S8: Time dependent antibacterial activity measurement of chiral co-assembled hybrid hydrogels LPH-EGCG and DPH-EGCG against (a) *S. aureus* and (b) *E. coli* at OD 600 nm; Figure S9: (a) Photographs of bacterial colonies formed by *S. aureus* and *E. coli* bacteria when treated with DPH-EGCG hybrid gel system with different content of EGCG. (b) corresponding rate of bacterial inhibition; Figure S10: SEM images exhibiting the morphology of bacteria cells (*S. aureus* and *E. coli*) before and after 5 h treatment with DPH-EGCG (scale bar: 3mm); Figure S11: Cell viability study of DPFEG, DPH-EGCG chiral hybrid systems when co-culture with HUVEC cells for 24 and 48 h; Figure S12: Quantitative measurements of cell adhesion of HUVEC cells when cocultured with DPFEG and DPH-EGCG chiral hybrid systems. for 6 h, 24 h, 48 h, and 72 h incubation time period; Figure S13: Fluorescence microscope images of live and dead cells of HUVEC after cultured for different incubation time period with control (PS), DPFEG, and various chiral hybrid DPH-EGCG systems. (Scale bars: 100 mm); Figure S14: Quantitative measurements of relative cell scratch area measured in control and chiral hybrid groups after 0, 12, and 24 h incubation time; Figure S15: Images of HUVEC cell scratch areas observed at specific time intervals, when treated with various groups. (Scale bars: 50 mm); Table S1: Composition of chiral LPH-EGCG hydrogels; Table S2: Composition of chiral DPH-EGCG hydrogels; Table S3: DPPH and ABTS scavenging efficiencies exhibited by chiral co-assembled hydrogels; Table S4: Quantitative values of residual biofilm mass when treated with different hydrogel groups; Table S5: Quantitative measurements of rate of inhibition. Table S6: Quantitative measurements of diameter of inhibition zone (mm).
