# Supplementary material for: Supramolecular Polymer Co-Assembled Multifunctional Chiral Hybrid Hydrogels with Adhesive, Self-Healing and Antibacterial Properties
Source: Gels. 2024 Jul 24;10(8):489. doi: 10.3390/gels10080489 (PMC11354115; doi:10.3390/gels10080489)
Supplement: Supplementary file 1 [file gels-10-00489-s001.zip › gels-3093625-supplementary.pdf]

Supporting information

Supramolecular-polymer co-assembled multifunctional chiral hybrid hydrogels with adhesive, self-healing and antibacterial properties

Zakia Riaz, Sravan Baddi*, Fengli Gao, Xiaxin Qiu and Chuan Liang Feng*

State Key Lab of Metal Matrix Composites, Shanghai Key Laboratory for Molecular Engineering of Chiral Drugs, School of Materials Science and Engineering, Shanghai Jiaotong University, Dongchuan Rd 800, 200240, Shanghai, China.

* *Corresponding Author*: clfeng@sjtu.edu.cn, sravanbaddi@sjtu.edu.cn

S1. Experimental section

S1.1.Materials

Poly(vinyl alcohol) (PVA) alcoholysis degree>99.5 mol%, molecular weight ~130000, Hyaluronic acid (HA) (97%+, Mw 700k-1400k), and Epigallocatechin-3-gallate (EGCG) were purchased from Adamas Chemicals, Shanghai, China. 2,2'-azino-bis(3-ethylbenzothiazoline-6-sulfonic acid) diammonium salt (ABTS) and potassium persulfate ($K_2S_2O_8$) were procured from Macklin Biochemical (Shanghai) Co., Ltd. Similarly, 1,1-diphenyl-2-picrylhydrazyl (DPPH) was sourced from the same supplier. Additionally, L/D-phenylalanine methyl ester hydrochloride, triethylamine, terephthaloyl chloride, and various common solvents such as ethanol, methanol, dichloromethane, and hydrochloric acid were obtained from Adamas Chemicals, Shanghai, China. Rhodamine B was purchased from Adamas chemicals, Shanghai, China. All chemicals are used as purchased without additional purification.

S1.2.Synthesis of L/D-phenylalanine based (LPFEG and DPFEG) gelator

The synthesis of the L/D-phenylalanine-based gelator followed a previously reported procedure from the literature [1]. Terephthaloyl chloride (2.6 g, 13.0 mmol) was slowly dissolved in 20 mL of dichloromethane (DCM). Subsequently, under constant magnetic stirring, a mixture containing L-phenylalanine methyl ester hydrochloride (6.0 g, 26.2 mmol) and triethylamine (8.0 mL, 58.4 mmol) in 100 mL of solvent was slowly added and left to stir for 12h. Excess solvent was removed by rotary evaporation, and any remaining residue was dissolved in ethanol. After filtration and drying, the insoluble substance L/DPF methyl ester was obtained. This material (5.1 g, 10.5 mmol, 85%) was dissolved in methanol, resulting in a suspension. Then, a 15 mL aqueous solution of NaOH was slowly added dropwise with constant stirring until a clear solution was obtained. Continued stirring for 12h. The pH was

adjusted using 3.0 M HCl, resulting in the formation of gel-like precipitate. This precipitate was washed and filtered multiple times with water, yielding the L/DPF product (4.6 g, 9.9 mmol, 90%) after complete drying in a vacuum oven. The overall yield of the obtained product, L/DPF, was 76.5%. Next, the L/DPF product (4.6 g, 9.9 mmol, 90%) was dissolved completely in 50mL of diethylene glycol, and 0.5 mL of concentrated HCl was added carefully. The solution was continuously stirred for 4 hours at 130°C. The solution was then poured into ice-cold water resulting in the formation of gel-like precipitates. The filtrate obtained was washed multiple times with deionized water. After drying the residual product was further dried in a vacuum oven to obtain L/DPFEG gelator (4.2 g, 6.6 mmol, 91%).

The reaction scheme for formation of LPFEG and DPFEG hydrogels are as followed.

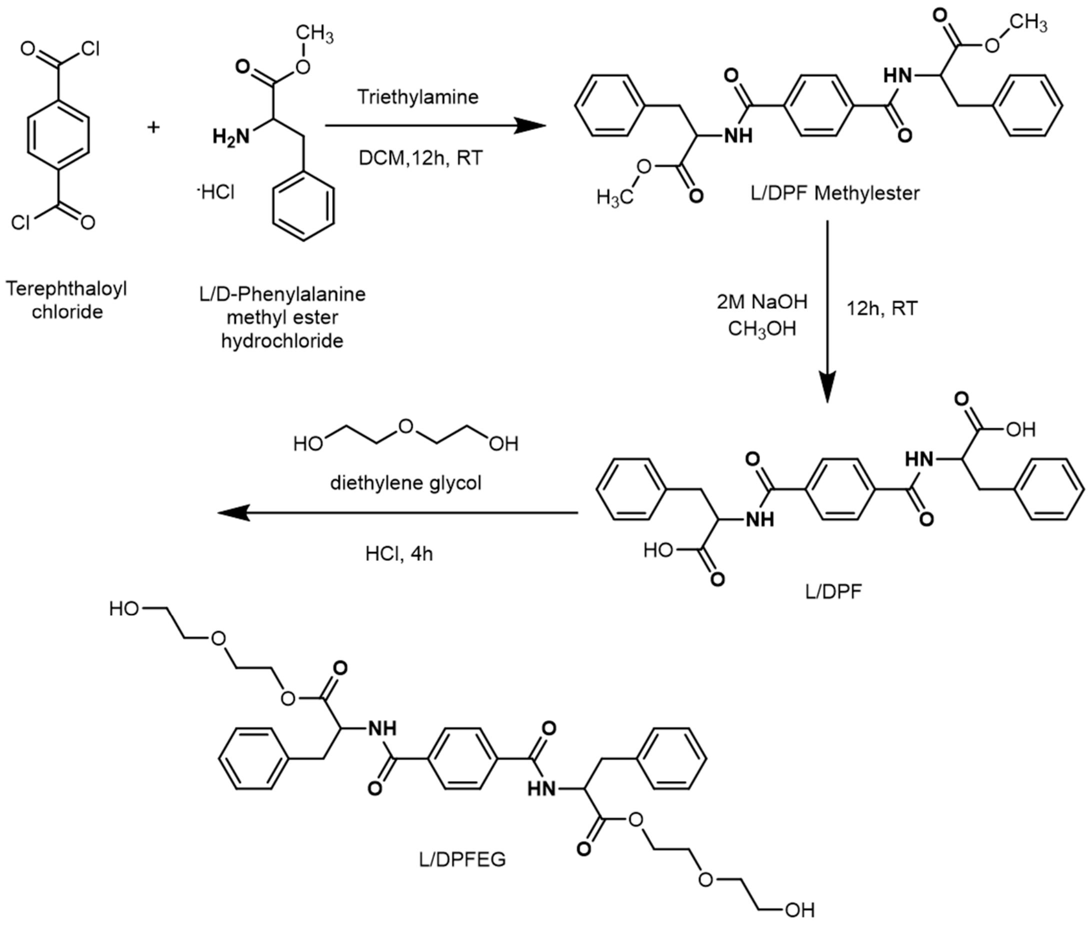

S1.3.Multiple free radicals scavenging procedure

The free radical scavenging activity was evaluated using the DPPH· scavenging method. Pure L/DPFEG hydrogel and chiral co-assembled L/DPH-EGCG hybrid hydrogels were tested by measuring the UV absorbance at 517 nm. Initially, DPPH was dissolved in ethanol solution at a concentration of 0.05 mg/mL. Approximately 2 mL of the DPPH solution was added to both

the pure hydrogel (3mg/mL) and the hybrid hydrogels (the same concentrations mentioned in table S1 & S2), and the resulting solution was left undisturbed in a dark place at room temperature for 30 minutes. Subsequently, the UV absorbance of the solution was measured. The DPPH free radical scavenging efficiency (*Y*) was calculated using the following equation (1).

$$Y = \left[\frac{(A_0 - A_n)}{A_o}\right] \times 100\% \qquad (S1)$$

Whereas, A_o and A_n indicates the measured UV absorbance of pure DPPH solution and DPPH with L/DPFEG and L/DPH-EGCG hybrid hydrogel solutions at 517 nm.

For ABTS assay initially the ABTS·+ radical was generated by oxidizing an ABTS solution with the addition of potassium persulfate ($K_2S_2O_8$). Subsequently, the same procedure as described above was employed, where pure L/DPFEG and chiral co-assembled hybrid hydrogels were combined with the ABTS·+ solution. After 30 minutes, the UV absorbance was measured at 734 nm. The corresponding free radical scavenging efficiency (*X*) was calculated using the equation (ii) provided below.

$$X = \left[\frac{(A_S - A_r)}{S}\right] \times 100\% \qquad (S2)$$

While A_s and A_r are corresponding UV absorbance values of pure ABTS. + solution and ABTS. + solution with LPFEG and DPFEG hydrogel and L/DPH-EGCG hybrid hydrogels with varying content of EGCG.

S2. Additional Experimental figures and tables

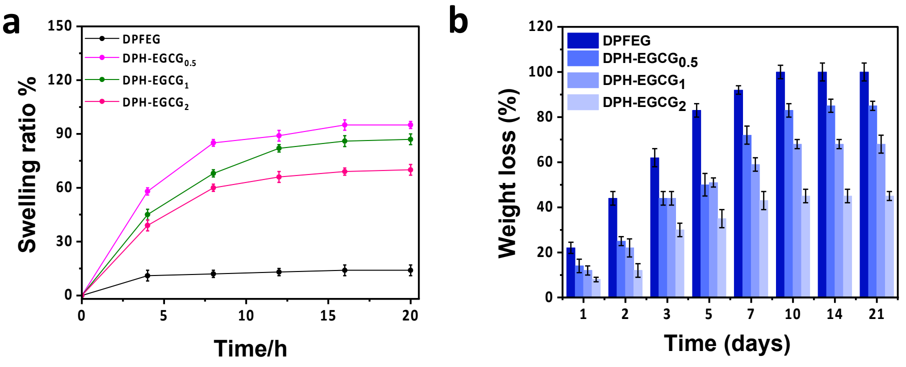

Figure S1. (a) Swelling ratio of prepared DPFEG and DPH-EGCG systems at selected time interval. (b) Weight loss profile of hydrogel samples in PBS at different days.

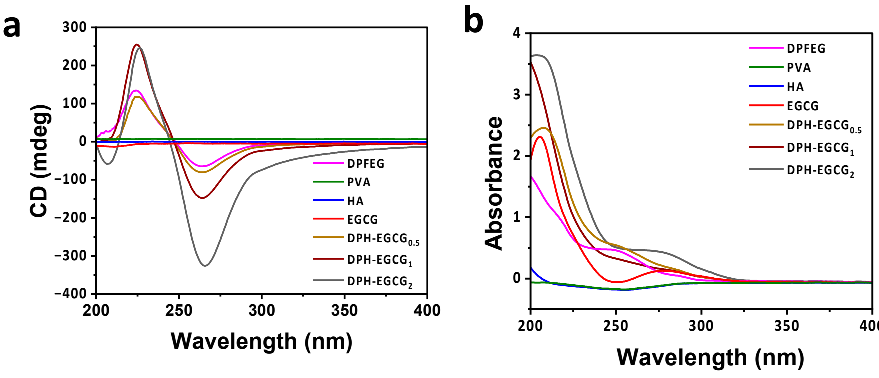

Figure S2. (a) CD spectra of neat DPFEG, PVA, HA, EGCG and DPH-EGCG hybrid chiral gels. (b) Showed their corresponding UV-vis spectra.

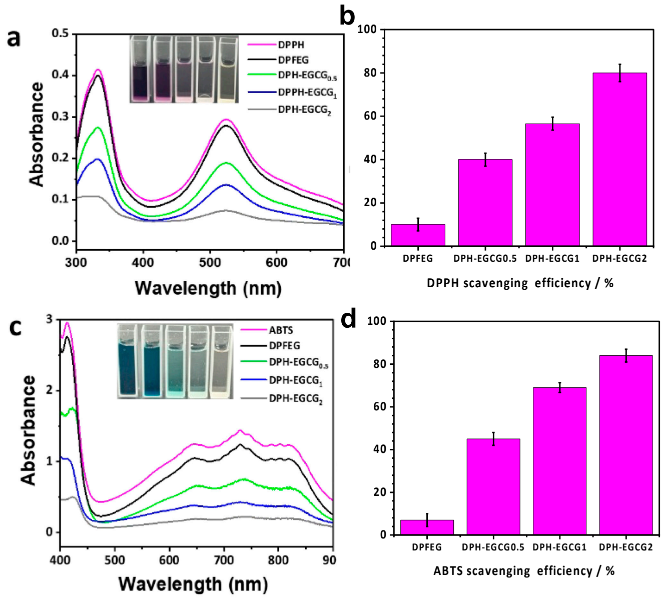

Figure S3. (a) UV-vis spectra present the absorbance of pure DPPH solution and change in their absorbance by addition of pure DPFEG, and DPH-EGCG hybrid hydrogels, while on top are images inserted showing change in their colours. (b) Scavenging effect of pure DPFEG and chiral hybrid hydrogels evaluated by DPPH scavenging assays (c) UV-vis spectra exhibiting the absorbance of neat ABTS solution and then change in its absorbance by addition of different groups naming DPFEG, and DPH-EGCG gels, while on top are images showing change in their corresponding solution colours. (d) ABTS scavenging efficiency of chiral hydrogels.

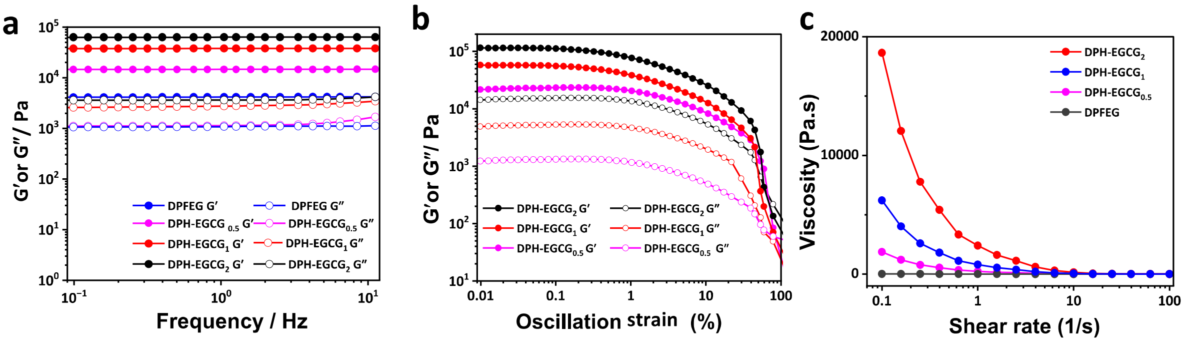

Figure S4. Rheological properties of neat DPFEG and chiral co-assembled hybrid hydrogels. (a) Frequency sweep test results at 25 °C. (b) Amplitude sweeps test measurements of gel systems at room temperature. (c) The shear viscosity profile at shear rate ranging from 0.1 to 100/s (a) pure DPFEG hydrogel and DPH-EGCG hybrid gel system at different concentration of EGCG.

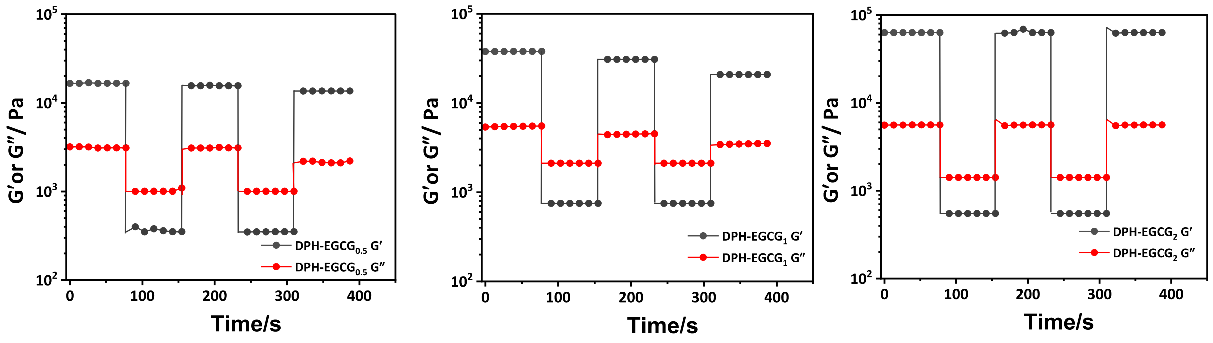

Figure S5. The self-healing properties of hybrid hydrogels obtained through continuous step-strain test at 25° C and fixed frequency.

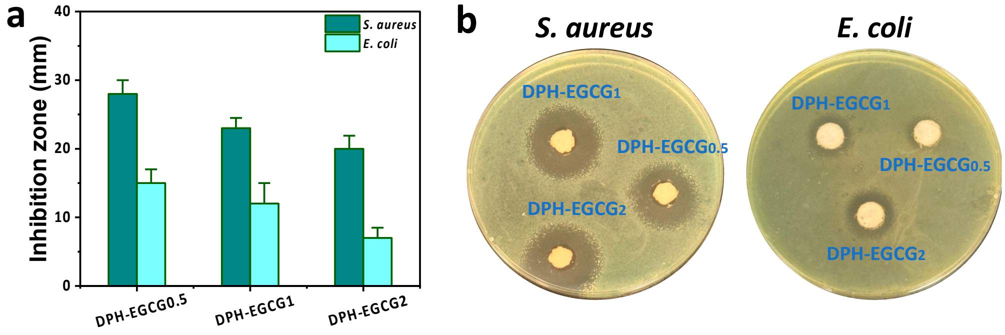

Figure S6. (a) Digital photos presenting the inhibition zone formed by DPH-EGCG hybrid gel against *S. aureus* and *E. coli*. (b) Corresponding quantitative measurements of diameter of zone of inhibition.

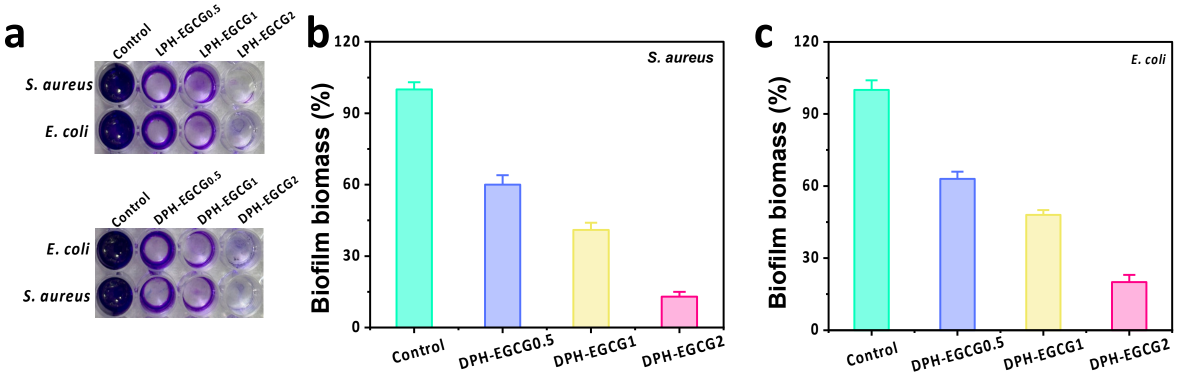

Figure S7. (a) Biofilm destruction and (b) and (c) corresponding measurement of biofilms biomass caused by DPH-EGCG chiral co-assembled hybrid hydrogels at varying loading of EGCG.

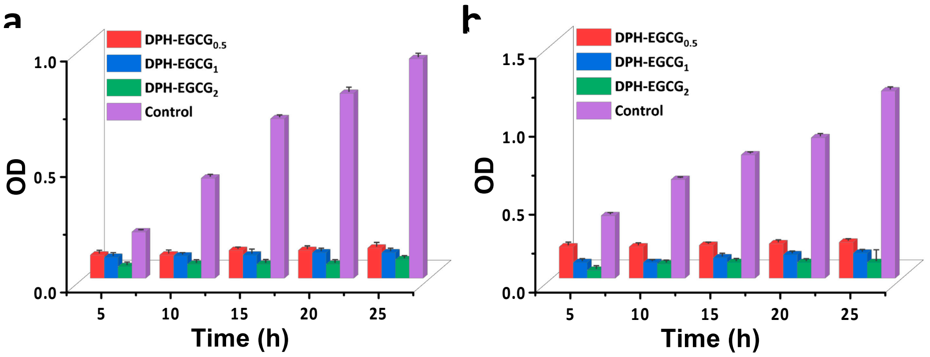

Figure S8. Time dependent antibacterial activity measurement of chiral co-assembled hybrid hydrogels LPH-EGCG and DPH-EGCG against (a) *S. aureus* and (b) *E. coli* at OD 600 nm.

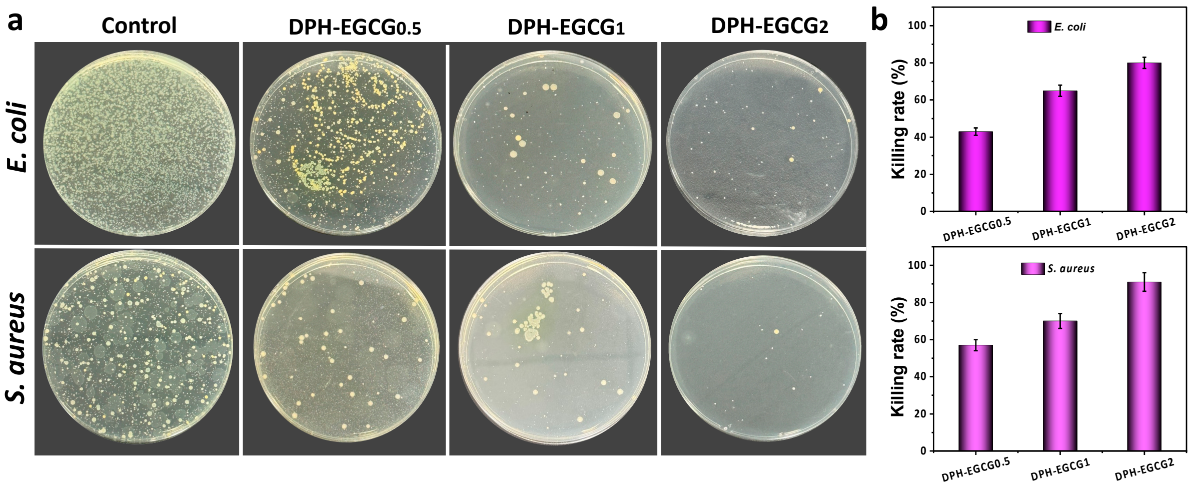

Figure S9. (a) Photographs of bacterial colonies formed by *S. aureus* and *E. coli* bacteria when treated with DPH-EGCG hybrid gel system with different content of EGCG. (b) corresponding rate of bacterial inhibition.

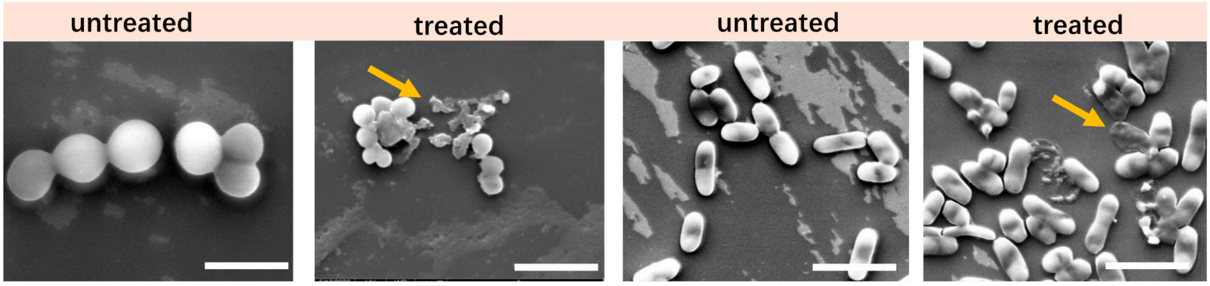

Figure S10. SEM images exhibiting the morphology of bacteria cells (*S. aureus* and *E. coli*) before and after 5 h treatment with DPH-EGCG (scale bar: 3μm).

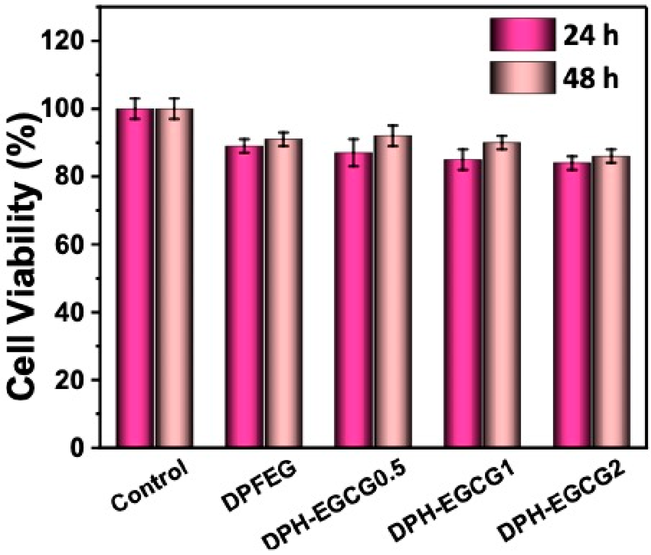

Figure S11. Cell viability study of DPFEG, DPH-EGCG chiral hybrid systems when co-culture with HUVEC cells for 24 and 48 h.

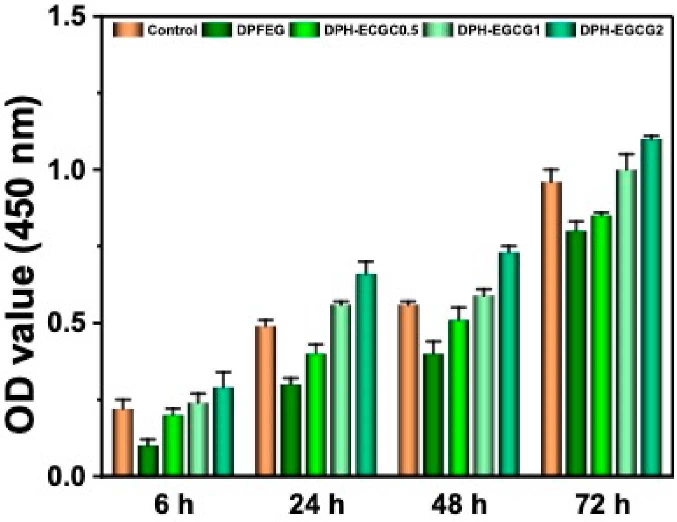

Figure S12. Quantitative measurements of cell adhesion of HUVEC cells when cocultured with DPFEG and DPH-EGCG chiral hybrid systems. for 6 h, 24 h, 48 h and 72 h incubation time period.

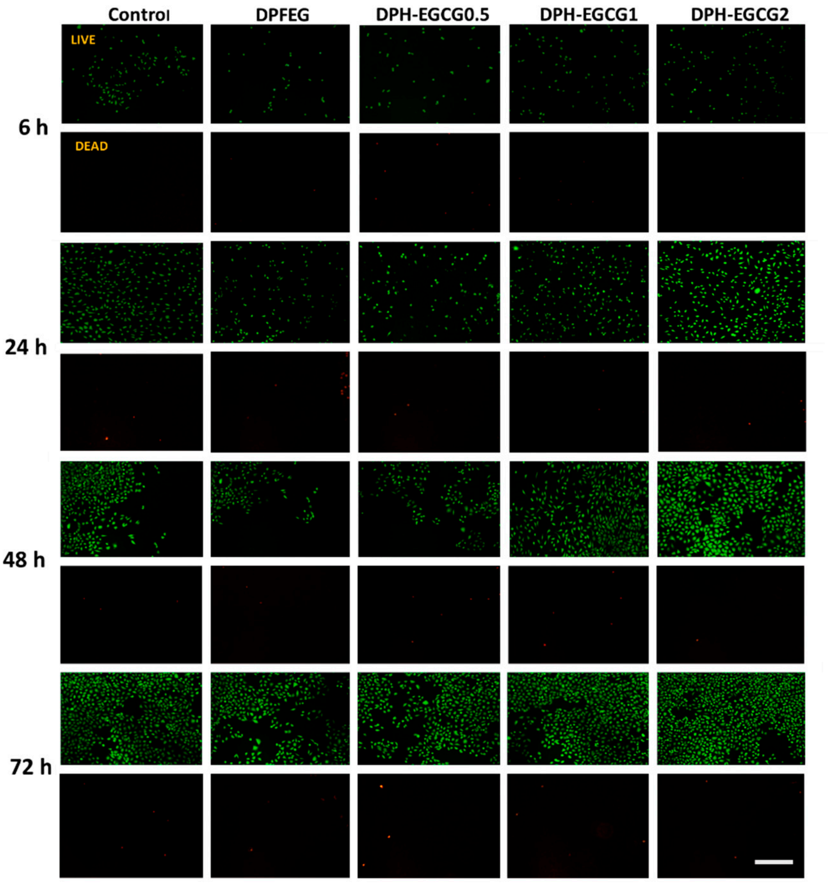

Figure S13. Fluorescence microscope images of live and dead cells of HUVEC after cultured for different incubation time period with control (PS), DPFEG, and various chiral hybrid DPH-EGCG systems. (Scale bars: 100 μm).

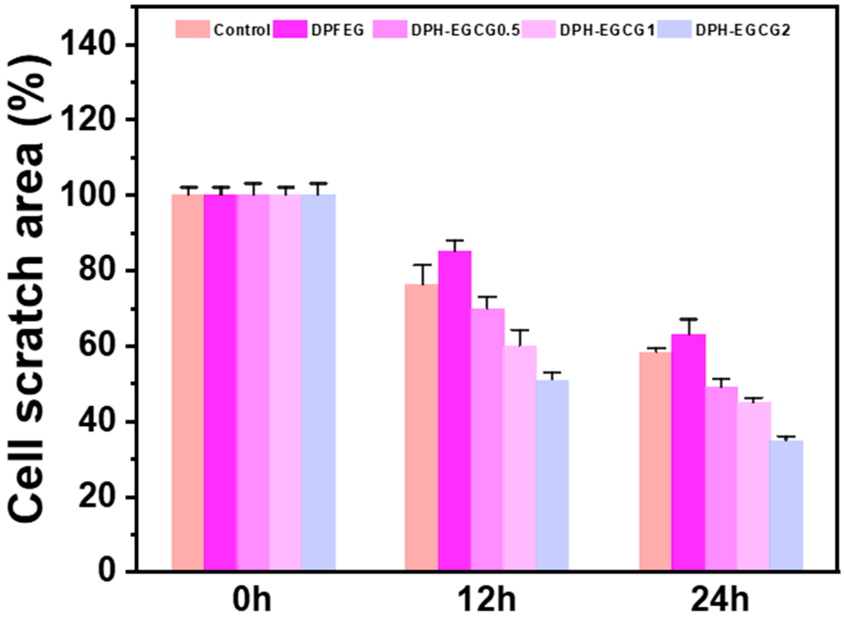

Figure S14. Quantitative measurements of relative cell scratch area measured in control and chiral hybrid groups after 0, 12 and 24 h incubation time.

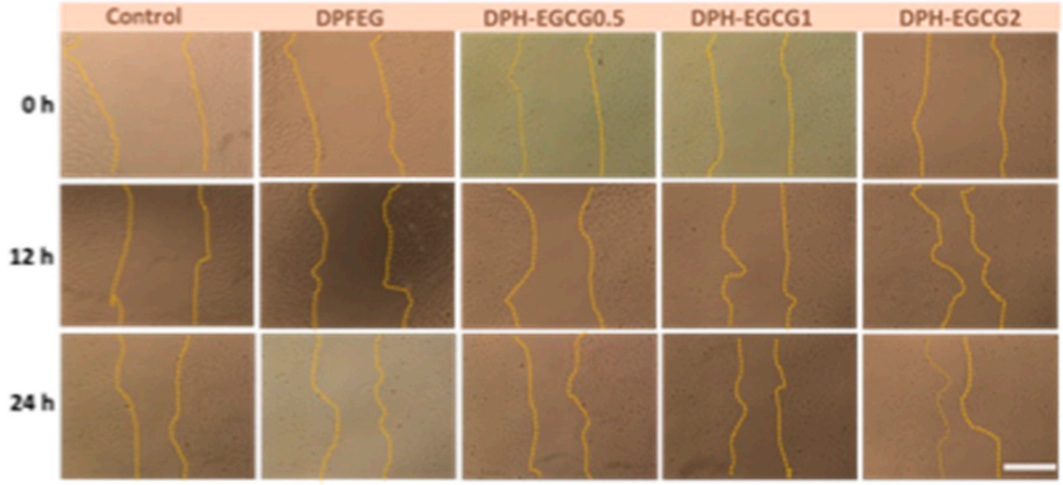

Figure S15. Images of HUVEC cell scratch areas observed at specific time intervals, when treated with various groups. (Scale bars: 50 µm).

Table S1. Composition of chiral LPH-EGCG hydrogels.

Name	PVA (mg/mL)	HA (mg/mL)	LPFEG (mg/mL)	EGCG (mg/mL)
LPH-EGCG$_{0.5}$	50	10	3	0.5
LPH-EGCG$_{1}$	50	10	3	1
LPH-EGCG$_{2}$	50	10	3	2

Table S2. Composition of chiral DPH-EGCG hydrogels.

Name	PVA (mg/mL)	HA (mg/mL)	DPFEG (mg/mL)	EGCG (mg/mL)
DPH-EGCG$_{0.5}$	50	10	3	0.5
DPH-EGCG$_{1}$	50	10	3	1
DPH-EGCG$_{2}$	50	10	3	2

Table S3: DPPH and ABTS scavenging efficiencies exhibited by chiral co-assembled hydrogels.

Hybrid hydrogel name	DPPH scavenging effect (%)	ABTS scavenging effect (%)
LPH-EGCG$_{0.5}$	50 ± 3	56 ± 3.6
LPH-EGCG$_{1}$	69 ± 2.3	75.5 ± 3.5
LPH-EGCG$_{2}$	90 ± 3	90 ± 2.9
DPH-EGCG$_{0.5}$	40 ± 3.2	45.3 ± 4
DPH-EGCG$_{1}$	56.6 ± 4	69.7 ± 2.3
DPH-EGCG$_{2}$	80 ± 3.1	84.1 ± 3.1

Table S4: Quantitative values of residual biofilm mass when treated with different hydrogel groups.

Hybrid hydrogel name	*S. aureus biofilms mass (g)*	*E. coli biofilm mass(g)*
LPH-EGCG0.5	60 ± 3.1	59 ± 4
LPH-EGCG1	39 ± 3.6	44 ± 3.9
LPH-EGCG2	8 ± 1	16 ± 3
DPH-EGCG0.5	60 ± 4	63 ± 3
DPH-EGCG1	41 ± 3	48 ± 2
DPH-EGCG2	13 ± 2.1	20 ± 3.2

Table S5: Quantitative measurements of rate of inhibition.

Hybrid hydrogel name	*S. aureus*	*E. coli*
LPH-EGCG0.5	45 ± 3.1	51 ± 3.2
LPH-EGCG1	70 ± 3	73 ± 3
LPH-EGCG2	98 ± 4	99 ± 4.9
DPH-EGCG0.5	57 ± 3.2	43 ± 1.9
DPH-EGCG1	70 ± 4	65 ± 3
DPH-EGCG2	91 ± 5.1	80 ± 3.4

Table S4: Quantitative measurements of diameter of inhibition zone (mm).

Hybrid hydrogel name	*S. aureus*	*E. coli*
LPH-EGCG0.5	23 ± 2.1	10 ± 2
LPH-EGCG1	27 ± 2.3	14 ± 1.5
LPH-EGCG2	31 ± 1.5	17 ± 2
DPH-EGCG0.5	20 ± 1.9	7 ± 1.5
DPH-EGCG1	23 ± 1.5	12 ± 3
DPH-EGCG2	28 ± 2	15 ± 2

Reference

1. Riaz, Z.; Baddi, S.; Gao, F.; Feng, C.-L. Mxene-Based Supramolecular Composite Hydrogels for Antioxidant and Photothermal Antibacterial Activities. *Macromol. Biosci.* **2023**, 2300082, doi:10.1002/mabi.202300082.
